# A comprehensive view on r-protein binding and rRNA domain structuring during early eukaryotic ribosome formation

**DOI:** 10.1093/nar/gkag036

**Published:** 2026-01-22

**Authors:** Magdalena Gerhalter, Michael Prattes, Lorenz Emanuel Grundmann, Irina Grishkovskaya, Enrico F Semeraro, Gertrude Zisser, Harald Kotisch, Juliane Merl-Pham, Stefanie M Hauck, David Haselbach, Helmut Bergler

**Affiliations:** Institute of Molecular Biosciences, University of Graz, Graz 8010, Austria; Institute of Molecular Biosciences, University of Graz, Graz 8010, Austria; BioTechMed-Graz, Graz 8010, Austria; Research Institute of Molecular Pathology (IMP), Vienna BioCenter, Vienna 1030, Austria; Vienna BioCenter PhD Program, Doctoral School of the University of Vienna and Medical University of Vienna, 1030 Vienna, Austria; Research Institute of Molecular Pathology (IMP), Vienna BioCenter, Vienna 1030, Austria; Institute of Molecular Biosciences, University of Graz, Graz 8010, Austria; BioTechMed-Graz, Graz 8010, Austria; Field of Excellence BioHealth, University of Graz, Graz, Graz 8010, Austria; Institute of Molecular Biosciences, University of Graz, Graz 8010, Austria; Research Institute of Molecular Pathology (IMP), Vienna BioCenter, Vienna 1030, Austria; Metabolomics and Proteomics Core, Helmholtz Center Munich, German Center for Environmental Health GmbH, D-80939, Germany; Metabolomics and Proteomics Core, Helmholtz Center Munich, German Center for Environmental Health GmbH, D-80939, Germany; Research Institute of Molecular Pathology (IMP), Vienna BioCenter, Vienna 1030, Austria; Institute of Molecular Biosciences, University of Graz, Graz 8010, Austria; BioTechMed-Graz, Graz 8010, Austria; Field of Excellence BioHealth, University of Graz, Graz, Graz 8010, Austria

## Abstract

Formation of the eukaryotic ribosomal subunits follows a strict regime to assemble ribosomal proteins (r-protein) with ribosomal RNAs (rRNA) while removing internal (ITS) and external (ETS) transcribed rRNA spacers. During the early stages of large subunit (LSU) formation, ITS2, together with six assembly factors, forms the characteristic foot structure of early nuclear pre-LSU particles. Here, we address the function of this foot structure during the early stages of ribosome assembly. We present cryo-EM structures from wild-type cells and cells depleted for the foot structure factor Rlp7. We show that compaction of domain I of the 25S rRNA is strictly dependent on the presence of foot factors, while domain II folds independently. Furthermore, Rlp7-depletion accumulated small subunit (SSU) processome intermediates prior to A1 cleavage and compaction of the individual domains of the 18S rRNA, providing also novel insights into the SSU-assembly process. SILAC labeling and affinity purification of co-transcriptionally assembled pre-ribosomes enabled us to resolve the assembly line of most early binding r-proteins step by step. This showed that incorporation of r-proteins in eukaryotes neither follows the bacterial regime nor a strict linear co-transcriptional mode. Instead, seed r-proteins might structurally define the individual rRNA domains before their compaction and fixation in the context of early pre-ribosomes.

## Graphical Abstract

**Graphical Abstract:**
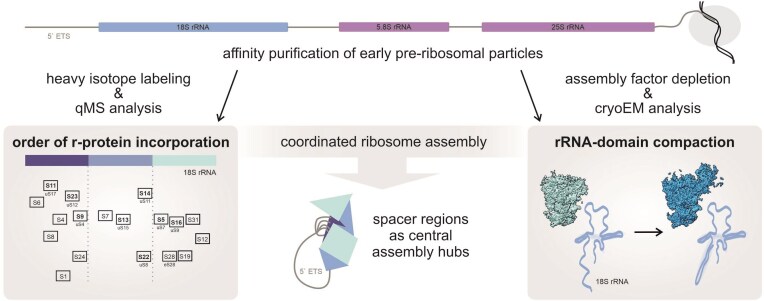

## Introduction

Eukaryotic ribosomes are composed of a large 60S (LSU) and a small 40S (SSU) subunit, both containing ribosomal RNAs (rRNAs) and ribosomal proteins (r-proteins). The formation of ribosomes is best understood in *Saccharomyces cerevisiae*, where RNA polymerase I transcribes a long 35S precursor rRNA (pre-rRNA) that contains the sequences of 3 of the 4 mature rRNAs (18S rRNA (SSU), 25S, and 5.8S rRNA (LSU)) (Supplementary Fig. S1A).

On this 35S precursor, the rRNAs are separated by internal (ITS) and flanked by external transcribed spacers (ETS), which are removed during the maturation process by endonucleolytic cleavage and exonucleolytic trimming reactions (Supplementary Fig. S1A). Cleavage at site A2 within the ITS1 separates the 20S pre-rRNA-associated SSU precursor from the 27SA_2_ pre-rRNA-associated LSU precursor. The latter is rapidly processed to form the 27SA_3_ and 27SB pre-rRNA during nucleolar LSU maturation. Further processing steps in the nucleoplasm and cytoplasm form the mature 25S and 5.8S rRNA. To convert the 20S pre-rRNA into the mature 18S rRNA, it is cleaved in the cytoplasm (Supplementary Fig. S1A) (reviewed in [1–3]).

Along with the stepwise processing of the (pre)-rRNAs, around 80 r-proteins are gradually incorporated into the nascent rRNA scaffold. While many r-proteins join early in the nucleolus, the last are incorporated only in the cytoplasm [4, 5]. The rRNA domains thereby gradually compact and adopt their mature secondary and tertiary structures that are stabilized by the incorporated r-proteins. The whole assembly process is assisted by more than 250 assembly factors that catalyze pre-rRNA cleavage and processing, stabilize intermediate states, and ensure timely orchestration of the individual maturation events (reviewed in [6–8]).

The SSU processome, also designated as 90S particle, is formed by modular building blocks that co-transcriptionally assemble with the 35S pre-rRNA ([9] reviewed in [10]). The process is initiated by co-transcriptional loading of the U three protein complex A (UTP-A) onto the nascent 5` ETS. Subsequently the UTP-B complex joins together with the U3 snoRNP (small nucleolar ribonucleoprotein) and finally the UTP-C complex is recruited. The U3 snoRNP stably associates by base pairing with different regions within the 5` ETS and the nascent 18S rRNA (Supplementary Fig. S1B). Further assembly reactions lead to the fully established SSU processome that contains more than 50 assembly factors and approximately 17 r-proteins. Cleavage in site A1 triggers disassembly of the SSU processome. This liberates an advanced small subunit precursor (also pre-40S) that, after additional cleavage in site A2, is rapidly exported into the cytoplasm, where the last maturation steps take place ([11, 12]; reviewed in [7, 13]).

The first LSU-specific factors and r-proteins also bind the emerging 35S pre-rRNA that is subsequently compacted in the 25S rRNA domains I and II [14, 15]. The ITS2 thereby serves as a binding site for six LSU assembly factors to form the foot structure characteristic of early pre-LSU particles that is only removed during nucleoplasmic stages of maturation.

Ground-breaking work by different laboratories highlighted many key events of large and small subunit maturation, including the SSU processome by cryo-EM ([16, 17, 14, 18–23] reviewed in [13]). While there are studies visualizing the SSU processome to pre-40S transition, there is little information to fully retrace the assembly of the earliest SSU processome as well as LSU precursors. Studies using truncated pre-rRNAs as scaffolds for particle assembly provided the first insights into initial binding events. Overexpression of these (pre)-rRNA mimics pointed towards a linear co-transcriptional assembly of the 18S rRNA domains [9, 24]. According to this model, parts that are transcribed first are assembled prior to later transcribed regions. This strategy also yielded the structure of the 5` ETS RNP, which was assembled readily in the absence of the full downstream 18S rRNA sequence [25] as well as SSU processome intermediates that contain the 18S rRNA domains largely assembled. However, recent cryo-EM structures from the thermophilic fungus *Chaetomium thermophilus* revealed SSU processome particles with a fully folded central domain, whereas the upstream 5` domain is stably folded only at a later stage. This challenged the view of a strictly linear incorporation of the rRNA domains [26].

Here, we present a series of cryo-EM structures of very early pre-ribosomes obtained after depletion of the pre-LSU maturation factor Rlp7. Our data show that a fully established foot structure is crucial for 25S rRNA domain I compaction, while domain II is folded independently. In addition, we identified three early SSU processome intermediates. While the body of these SSU processome states is already stable and well resolved, these states show clear differences in the head and platform region. The least assembled state has neither a stably assembled 5` apical subdomain nor an incorporated central domain. To better understand the very early steps of ribosome formation, we traced the incorporation of r-proteins by stable isotope labeling by amino acids in cell culture (SILAC), which proved to be an extremely powerful tool to dissect the assembly order of most early binding r-proteins step by step. These data show that eukaryotic r-proteins are incorporated in a different order than their bacterial counterparts and do not follow a strict linear co-transcriptional incorporation scheme. Instead, early binding seed r-proteins initially define domains of the rRNA that are only later compacted and finally locked in the context of the pre-ribosome by later binding r-proteins.

## Material and methods

### Statistics, reproducibility, and integrity

All pre-ribosomal particle purifications, including subsequent SDS-PAGE, western blot, and northern blot analysis (Main and Supplementary Figures), were independently repeated at least twice with the same output. For the SILAC experiment as well as the polysome profile with subsequent analysis, three independent experiments were conducted.

Uncropped gels and blots for all figures and supplementary figures are shown in the source data file. For each western or northern blot, one single membrane was used to consecutively detect all antibodies or probes. For western blot analysis of the polysome profiles, the same samples had to be loaded on several membranes, but three antibodies were detected on all membranes to verify identical loading. The source data includes an additional Excel sheet with the calculations for the qMS SILAC analysis.

## Biochemical methods

### Strains and plasmids

All yeast strains used in this study are listed in Supplementary Table S1. Chromosomal gene fusions were generated by homologous recombination with linear PCR products to transform the corresponding yeast strain as described recently [27, 28]. All Plasmids and primers used for strain construction in this study are listed in Supplementary Table S2.

### Cultivation P*_GAL1_* strains and SILAC

#### Cultivation P_GAL_-Rlp7

Each P*_GAL1_*-Rlp7 strain, having different TAP-tagged bait proteins (Noc2, Nop7, Bud20), was grown in 500 mL YPGal to a late logarithmic phase. Each culture was then split and transferred into 2 liters of YPD and 2 liters of YPGal, allowing logarithmic growth for 16 h prior to harvesting.

#### Medium and cultivation Utp7-TAP for SILAC

Minimal medium with CSM-Lysin (MP Biomedicals) was prepared and equally split into separate flasks. Half of the flasks were additionally supplied with unlabeled L-Lysine (Silantes; light medium), while the other half was supplied with L-Lysine ^13^C ^15^N labeled (98% purity, Silantes; heavy medium). The preparatory Utp7-TAP culture was transferred to light medium to reach late exponential growth. Cells were harvested at 6000 x g for 5 min at 25°C. For the 3 min ^13^C ^15^N lysine labeling, the cell pellets were resuspended in heavy medium (pre-warmed) and harvested immediately in the very same bucket. For the 15 min ^13^C ^15^N lysine labeling, cell pellets were resuspended in heavy medium and incubated on the shaker for 12 min prior to harvesting.

#### Harvesting

Cells were harvested in a Beckmann centrifuge at 6000 x g for 3 min at 4°C using the JLA 81 000 rotor. The cell pellets were washed with ice-cold water and centrifuged at 1250 x g for 10 min at 4°C.

### Affinity purification of TAP strains

*Purification* - First, cell pellets were resuspended in one volume of lysis buffer with protease inhibitors (20 mM HEPES, 10 mM KCl, 2.5 mM MgCl_2_ and 1 mM EGTA, pH 7.5 with 1 mM DTT, 0.5 mM PMSF and 1 x FY-protease inhibitor (Serva)) and 1.5 volumes of 0.6 mm glass beads (Sartorius AG) was added to break cells for 4 min in a bead mill (Merkenschlager). Dissoluble debris was removed by centrifugation at 4°C at 41 000 x g for 30 min. 20 µL aliquots for protein and RNA analysis were taken from the resulting crude extract. To isolate pre-ribosomal particles, the crude extract was incubated with self-coupled magnetic IgG beads for 90 min [29, 30, 5]. Lysis buffer with 1 mM DTT was used for five washing steps, after which 1/5 of the bead volume was removed and stored for RNA analysis (= pull-down). Pre-ribosomal particles were eluted overnight with self-made TEV-protease treated with RiboLock RNase Inhibitor (Thermo Fisher) in lysis buffer with 100 mM NaCl and 1 mM DTT. The resulting TEV-eluates were analyzed by SDS-PAGE, western blots, and further used for plunge freezing with subsequent cryo-EM data acquisition.

### SDS-PAGE and Western Blot analysis

Protein samples were separated on precast 4–12% NuPAGE gels (Novex Life Technologies) and blotted on a PVDF membrane (Carl Roth GmbH) using the tank-blot system (Hoefer). Chemiluminescence signals were detected using the Clarity™ Western Blotting Detection Reagent (Bio-Rad) and the ChemiDoc™ Touch Imaging System (Bio-Rad). All antibodies are listed in Supplementary Table S3.

### RNA isolation and Northern blotting

#### RNA isolation

RNA was extracted from crude extract or pull-downs of the above-described TAP purifications. Two rounds of phenol-chloroform-isoamylalcohol (25:24:1) extraction, followed by two times chloroform-isoamylalcohol (24:1) extraction and ethanol precipitation, were performed.

#### Northern Blot

Around 2 μg of crude extract RNA or undiluted pull-down RNA were separated in 1.5% agarose gels. The separated RNA was transferred onto a Hybond-N nylon membrane (Amersham Biosciences) overnight.

#### Radioactive detection

5′-^32^P-labeled oligonucleotide probes in 7% SDS, 1 mM EDTA, 500 mM NaPO_4_ buffer [pH 7.2] were hybridized overnight at 42°C (EC_2_ 37°C) to the Northern Blot membrane (Supplementary Fig. S1A, Supplementary Table S4). After washing three times for 20 min at 42°C in 1% SDS, 40 mM NaPO_4_ buffer [pH 7.2], X-ray films were exposed. Probes were stripped from membranes using 1% SDS four times for 15 min.

### Polysome profile analysis

#### Cultivation and cell lysate

The Noc2-TAP P*_GAL1_*-Rlp7 strain was cultivated in YPGal followed by 16 h shift to glucose or galactose (250 mL media) in the logarithmic growth phase. 50 µg/mL Cycloheximide was added, and cultures were incubated in an ice bath for 5 min. Cells were harvested with 3000 x g for 5 min at 4°C in a Beckmann centrifuge (JA-10 rotor). The breaking buffer (10 mM Tris, 100 mM NaCl, 30 mM MgCl_2_, pH 7.4, 50 µg/mL Cycloheximide, 1x Complete Protease-Inhibitor (Roche)) was used to wash the cell pellets once. Prior to breaking the cell pellet, it was dissolved in 1 time its volume with Breaking Puffer, and 1.5 times its volume of glass beads was added. Periodic shaking using a vortex was followed by centrifugation steps (10 min 5600 x g, 4°C, and two times 10 min 18 000 x g, 4°C). The OD_260_ of the crude extract was measured, and 100 OD_260_ units were used for subsequent sucrose centrifugation.

#### Sucrose centrifugation

Gradients were prepared as followed: Five steps of 10% to 50% sucrose were solved in 50 mM NH_4_Cl, 12 mM MgCl_2_, 50 mM Tris, 1 mM DTT pH 7.4. Starting with 50% sucrose, each step was allowed to freeze at -80°C for a minimum of 4 h before the next sucrose solution was added. Before centrifugation, the gradients were defrosted overnight, 100 OD_260_ units were loaded, and centrifuged at 4°C for 2 h and 45 min at 210 000 x g.

#### Polysome profile recording and sample fraction collection

OD_260_ of the gradients was recorded by the ISCO system with subsequent collection of 12 fractions per gradient. Proteins were precipitated by a total of 10% TCA overnight at 4°C, centrifuged, washed, and resuspended in 2 x FSB prior to SDS page and western blotting.

## Cryo-electron microscopy

### Plunge-freezing

Around 4 µl sample was added onto pre-floated Quantifoil 3.5/1 grids (2nm carbon) that were glow-discharged for 60 s using an ELMO system beforehand. The sample was incubated on the grids for 60 s at 4°C and 75% humidity in the automatic plunge freezer EM GP1 (Leica). After frontside plotting for 2 s, the grids were plunged into liquid ethane. Grids were stored in liquid nitrogen until data collection.

### Cryo-EM imaging settings

CryoEM data were collected in a 300 kV Titan Krios G4i equipped with a cold field emission gun, a Selectrus energy filter with 2 eV slit width, and a Falcon 4 direct electron counter. Micrographs were collected in EER format (40x fractionated) at a pixel size of 0.7441 (81 000 x magnification) and a cumulative dose of 40 e/Å^2^ using a defocus range of -2.0 to -4.0 µm in 0.25 increments. Automation of data collection was performed using EPU (Thermo Fisher).

### Image processing

Image processing was performed in Cryosparc v3.0 [31]. Preprocessing included patch motion correction and patch CTF estimation. Micrographs were excluded from further processing using the manually curate exposure job based on motion and CTF statisitics and ice contaminations and/or devitrification.

For the Noc2-TAP non-depleted sample, 9787 curated micrographs were used for particle picking. Initial 2D class averages generated from manually picked particles. These averages of the pre-LSU complex were used for template picking in both datasets. After multiple rounds of 2D classification, 124 777 particles were used to generate an initial 3D model (*ab initio*). Subsequently, multiple rounds of heterogeneous refinement, as well as 3D variability analysis followed by clustering, were performed to separate pre-ribosomal particle populations. This resulted in the identification of three different structures resembling published states in a resolution of 2.8–3.4 Å [18, 32].

For the Noc2-TAP Rlp7-depleted sample, 11 163 curated micrographs were used for particle picking. Initial 2D class averages generated from manually picked particles resulted in the identification of a smaller structure, also obvious in the initial 2D classification, that was identified to represent very early pre-LSU. These 2D classes were used as templates for particle picking in cryoSPARC. The remaining picks after 3D classification were used to train the Topaz picker [33]. The subsequently extracted 281 078 particles were then subjected to iterative 2D classification and heterogeneous refinement, resulting in 54 106 particles that were refined using NU-refinement. Finally, the map was post-processed using DeepEMhancer and the pretrained ‘high-res’ model provided by the developers (citation see below).

Moreover, one large structure representing an SSU processome (27 552 particles) was present in this dataset. Conformational heterogeneity in the refined particle population was assessed using the 3D variability analysis tool in Cryosparc v3.0 [34], which resulted in the identification of three assembly states (A1, A2, and A3 resolving to 3.2, 3.7, and 3.4Å, respectively). After the homogeneous refinement, the three averages were post-processed using DeepEMhancer as described above [35].

### Model fitting and model building

Initial rigid body fitting of individual chains of the models: PDB 8E5T for pre-LSU Rlp7-depleted; PDB 5WLC for 90S Rlp7-depleted state A1 and A2, PDB 5WLC and 6LQO for 90S Rlp7-depleted state A3; into the electron density was done with UCSF ChimeraX v1.7.1 [36]. Refinement of the models was done using Phenix v1.18.2–3874 [37] and Namdinator [38]. For molecular visualization, UCSF ChimeraX v1.7.1 was used [36]. The Rlp7-depleted pre-LSU was further modeled by iteratively using Isolde (v. 1.8) [39], to manually fix problematic regions, followed by phenix real space refinement (Supplementary Table S7).

An unknown density could be assigned to Nop4 by performing a Collabfold screen [40], predicting Noc1 against its known interactors, and comparing the high-confidence predictions manually against our cryoEM map.

### Mass spectrometric analysis

Three replicate datasets (two samples each) were analyzed by MS analysis (*n* = 3), resulting in a total of six analyzed samples. As described in detail above, the samples were TAP-eluates from the tandem affinity purifications, before which SILAC labeling with ^13^C ^15^N lysine was done. No unlabeled control was used.

### Proteomics sample preparation and LC-MSMS measurement

Proteins were digested with LysC and trypsin with a filter-aided sample preparation protocol (FASP) as described [41, 42]. Eluted peptides were acidified with TFA prior to data-dependent TOP15 LC–MSMS measurement on a QExactive HF-X mass spectrometer (Thermo Fisher Scientific) coupled online to a Ultimate 3000 RSLC nano-HPLC (Dionex) as described [43].

### Protein identification and label-free quantification of proteomic data

Acquired raw files were analyzed in the Proteome Discoverer software (version 2.5, Thermo Fisher Scientific) for peptide and protein identification and quantification as described [43], additionally allowing for the variable ^13^C ^15^N lysine (Lys-8) modification in the database search. Normalized abundance values of Lys-8 labeled and/or unlabeled peptides after filtering of identifications (XCorr score > 1 and peptide false discovery rate < 1%) were used for further calculations (see below).

### Data analysis (SILAC labeling)

The peptide list was filtered for ribosome biogenesis factors as well as r-proteins of the small and large subunits. The individual samples (3 or 15 min of labeling time with ^13^C ^15^N lysine) were analyzed as follows: For the total protein abundance, the abundance values of the peptides that correspond to one protein were summed up. On the one hand, these values were used to relate the total protein abundances of individual proteins to the abundance of the bait protein Utp7. On the other hand, these results were used to compare the composition of the particles of the individual samples among each other by mean and standard deviation of the individual proteins, if their relative abundance was at least 0.1 to the bait.

Next, the abundances of the peptides that include ^13^C ^15^N lysine that correspond to one protein were summed up; if one peptide included two ^13^C ^15^N lysine, we multiplied this abundance by two prior to forming the sum. The sum of abundances of ^13^C ^15^N lysine-containing peptides was then divided by the total protein abundance as described above (“labeling” within the main text of the manuscript). Next, the mean and error (half of the range) values of the three replicates of one sample (3 or 15 min of labeling time with ^13^C ^15^N lysine) were calculated. Some r-proteins showed zero-labeling in more than two of the six labeling values (three replicates, two timepoints) and also showed low abundance relative to the bait (<0.1) and therefore are most likely contamination and not part of a co-transcriptional pre-ribosomal particle. These proteins also showed high variability (relative errors > 50%) and are depicted by gray diamonds in Fig. 4A and B. They were omitted from Fig. 4C and D. The other ribosomal proteins from the 3 and 15 min samples showed a linear correlation (Fig. 4A and B). Data exhibited a non-zero intercept that is most likely due to a small delay in heavy-lysine labeling in the 3-min samples. The Pearson correlation coefficients for RPS and RPL r-proteins are ∼0.92 and ∼0.94, respectively. This enables us to combine the 3 and 15 min samples in a single data set to enhance the confidence of the combined results. To achieve that, we chose Rps11 (uS17) and Rpl8 (eL8) as references and normalized each data set by the respective labeling ratio of such proteins. These are the r-proteins with the largest difference between neighbor points and with the largest ^13^C ^15^N lysine relative content (Fig. 4). Finally, labeling ratios of the 3 and 15 min samples were combined, and mean and error values were calculated: (I) Mean values were obtained by a weighted average where the weights correspond to the respective precisions (inverse of the variance); (II) error values were retrieved by summing up precision values of the 3 and 15 min data sets.

## Results

### Rlp7 is required to establish a fully developed foot structure of pre-60S particles

**Figure 1.**
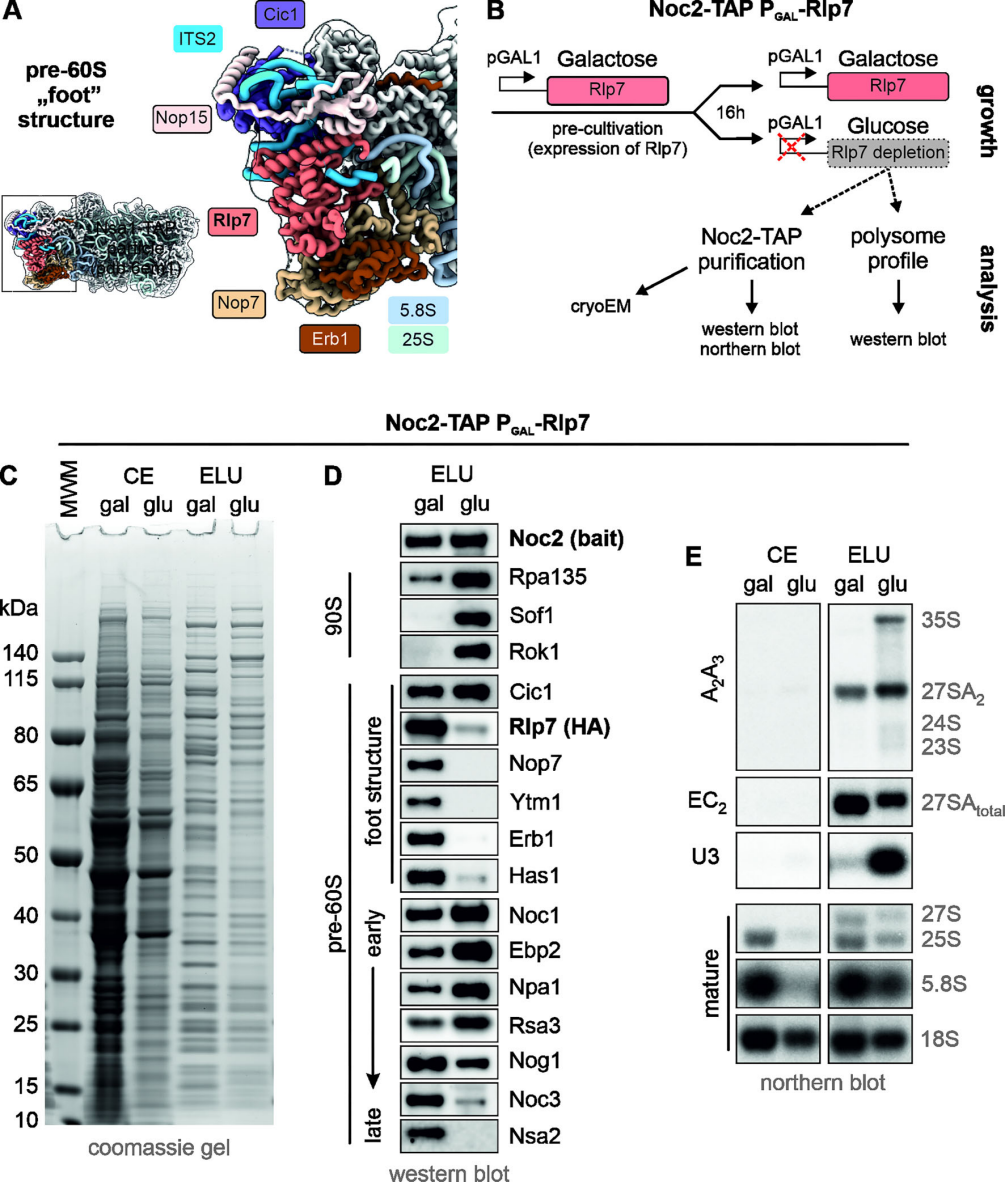
Rlp7 is required to fully establish the foot structure and its depletion rebounds to earlier steps in ribosome formation. (**A**) Overview of the pre-LSU assembly factors that bind around the ITS2 region to form the “foot” structure. (**B**) Cultivation timeline (growth) for the Noc2-TAP P_Gal1_-Rlp7 strain, resulting in a control sample grown in galactose (gal, wild-type conditions) and an Rlp7-depleted sample grown in glucose for 16 h (glu). (**C**) Following purification via Noc2-TAP, protein composition of the crude extract (CE) and the TEV eluate (ELU) was analyzed by SDS-PAGE. (**D**) The pre-ribosomal particles isolated via Noc2-TAP were analyzed by western blotting. (**E**) RNA isolated from the crude extract (CE) and the Noc2-TAP pull-down (ELU) was analyzed by northern blotting. The probes used are indicated on the left (Supplementary Fig. S1A), while the detected pre-rRNA species are labeled on the right.

To gain insight into which role the internal transcribed spacer 2 (ITS2) plays in the assembly process, we depleted the maturation factor Rlp7, which binds to the ITS2 spacer in a central position and is thus part of the characteristic foot structure of early nucleolar pre-60S particles (Fig. 1A). For this purpose, we used a strain expressing Rlp7 under the control of the repressible *GAL1* promoter [44, 45, 43]. Using Noc2-TAP as a bait protein allowed us to purify early co-transcriptional pre-ribosomal particles (Fig. 1B and C).

Depletion of Rlp7 resulted in reduced levels of mature LSU in polysome profiles (Supplementary Fig. S2B) and decreased levels of mature 25S and 5.8S rRNA characteristic of a large subunit formation defect (Supplementary Fig. S2A). In line with previous findings, depletion of Rlp7 led to a strong accumulation of early 27SA_2_ and 35S pre-rRNAs in RNA isolated from the Noc2-TAP pull-downs as well as from the crude extract (Fig. 1E and Supplementary Fig. S2A) [44, 45, 43]. Concomitantly, the U3 snoRNA, an integral part of the SSU processome, was observed in RNA isolated from the crude extracts and Noc2-TAP pull-downs upon depletion of Rlp7 (Fig. 1E and Supplementary Fig. S2A). These changes are in line with sedimentation of Noc2-TAP in the SSU processome range in sucrose gradient centrifugation (Supplementary Fig. S2B and C) and with accumulation of SSU processome factors (i.e. Sof1, Rok1) and early pre-LSU factors (i.e. Noc1, Npa1, Rsa3) in Noc2-TAP purifications (Fig. 1D). Furthermore, late joining pre-LSU maturation factors (Nsa2, Noc3) were depleted when Rlp7 was absent. Erb1, Nop7, and Ytm1 also failed to associate with the pre-ribosome (Fig. 1D). As these proteins sedimented in the soluble fraction of the sucrose gradient (Supplementary Fig. S2C) in the depleted strain, Rlp7 is required for recruitment of the heterotrimeric Erb1, Nop7, and Ytm1 complex to the ITS2-containing foot structure (Supplementary Fig. S3, Nop7-TAP samples) [46–48]. Later joining maturation factors (e.g. Bud20) cannot associate with Noc2-TAP particles and seemed to be degraded when Rlp7 was depleted (Supplementary Fig. S2C and S3 Bud20-TAP samples). Taken together, Noc2-TAP mainly co-purified very early pre-LSU particles as well as the SSU processome after Rlp7 depletion. The isolated SSU processomes represented early assembly stages prior to cleavage in A0 and A1, as obvious by the co-purification of only 35S pre-rRNA and not 33S or 32S pre-rRNA (Fig. 1E).

### A fully assembled foot is crucial for the compaction of 25S rRNA domain I of the LSU

To gain further insights into the function of the ITS2, we determined the structure of Noc2-TAP particles from the Rlp7-depleted and non-depleted strains by single particle cryo-EM (Supplementary Table S5 and Supplementary Fig. S4). The dataset of the non-depleted condition led to 3 particle populations, reflecting previously published states (Supplementary Figs S4 and S5A) [18, 32] confirming the biochemical data that the steady state of Noc2 pre-LSU population is at the 27SA_2_ to 27SB pre-rRNA stages (Fig. 1E). In line with the co-purifying rRNAs, we did not detect SSU processome particles in this cryo-EM dataset.

**Figure 2.**
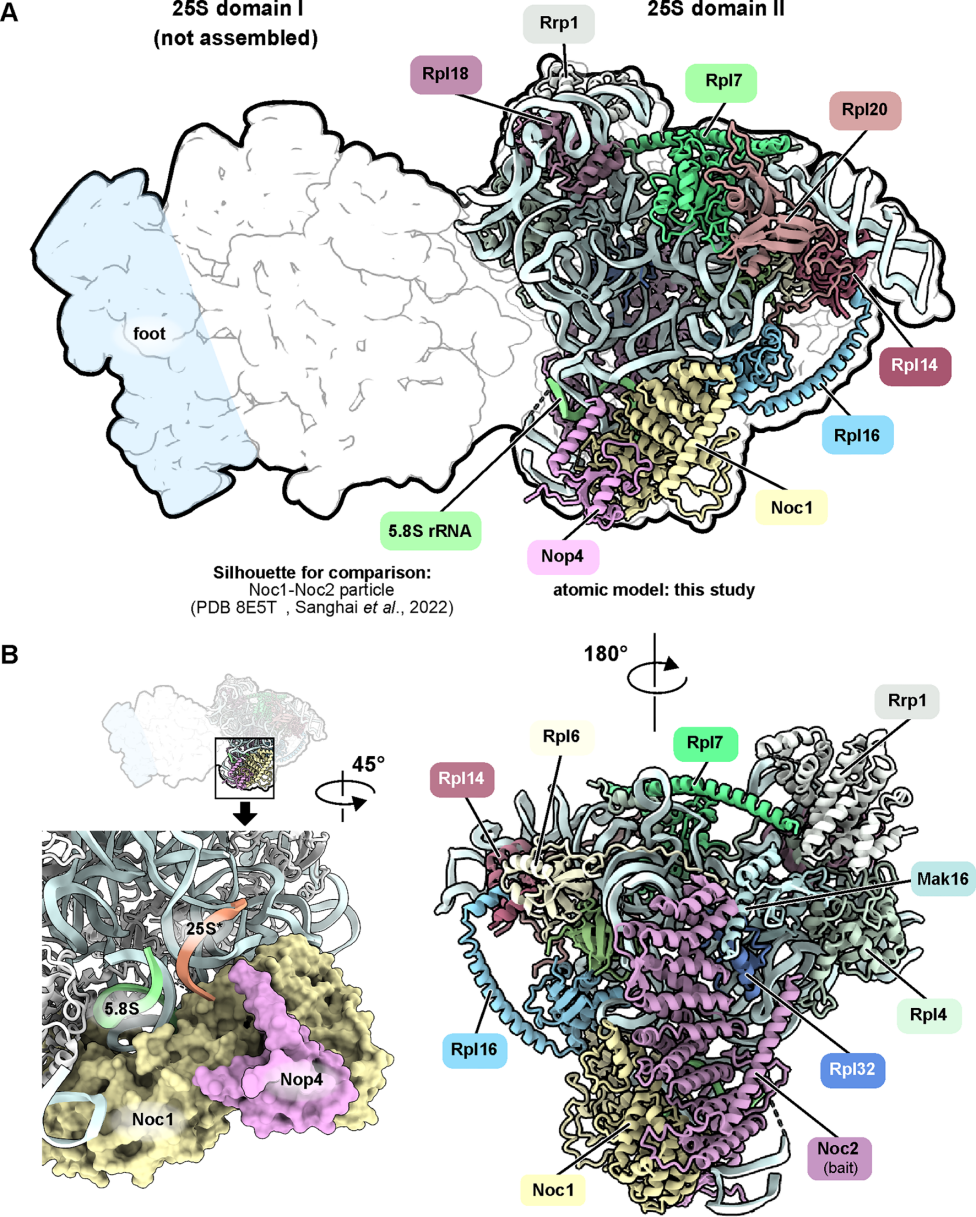
Depletion of Rlp7 prevents compaction of domain I and the foot structure. (**A**) The pre-LSU specific population of the Noc2-TAP particle after depletion of Rlp7 (Supplementary Fig. S4B) is depicted as a cartoon and superposed with the previously published co-transcriptional pre-LSU particle (PDB 8E5T, shown as a silhouette) isolated from rRNA mimics [15]. In the Rlp7-depleted particles, only 25S rRNA domain II is structured and resolved by cryo-EM, while domain I of the 25S rRNA and the foot structure (ITS2) are disordered and not visible in the reconstruction. (**B**) Detailed view of the Nop4-Noc1 interaction. The CRAC site of Nop4 near h26 of the 25S rRNA [50] is highlighted in orange (1436–1443 nt.), Noc1 (light yellow) and Nop4 (pink) are shown in surface representation.

Consistent with the western and northern blot results, cryo-EM analysis of Noc2-TAP particles purified after depletion of Rlp7 showed one very early pre-LSU particle population as well as three different SSU processome particle populations (Supplementary Fig. S4B). The single pre-LSU particle that we identified in this sample contains only the 25S rRNA domain II partially folded (Supplementary Fig. S5B, Supplementary Table S6 and S7). This domain is structured by the maturation factors Noc2, Noc1, Mak16, and Rrp1 and nine r-proteins (Fig. 2A) and closely resembles the domain II of a previously described very early co-transcriptional pre-LSU particle isolated from rRNA mimics [15]. In addition to these proteins, we observed a domain (residues 282–403) of the early nucleolar pre-60S assembly factor Nop4 [49]. Nop4 contains 4 RRM RNA-binding domains, and the domain we identified in our structure is adjacent to RRM3 and associated with Noc1 (Fig. 2B). It coincides with the Nop4 CRAC site near h26 of the 25S rRNA [50]. At this early stage of assembly, the four RRM domains of Nop4 likely serve to keep the still unstructured 25S rRNA in place.

In contrast to the published state, domain I and the ITS2 containing foot region are not structured in our particle and were not detected by masked local refinements or targeted picking using the structure showing both domains folded as a template. This shows that the foot structure is critical to compact the 25S rRNA domain I and confirms that the two domains can fold independently. As we still detected the domain I-specific assembly factor Ebp2 in TAP purifications from the depleted strain and the particle sediments in the LSU peak of the sucrose gradient (Fig. 1D and Supplementary Fig. S2C, glucose sample), the domain is at least partially assembled but not compacted.

### Very early SSU processome states accumulate upon Rlp7-depletion

**Figure 3.**
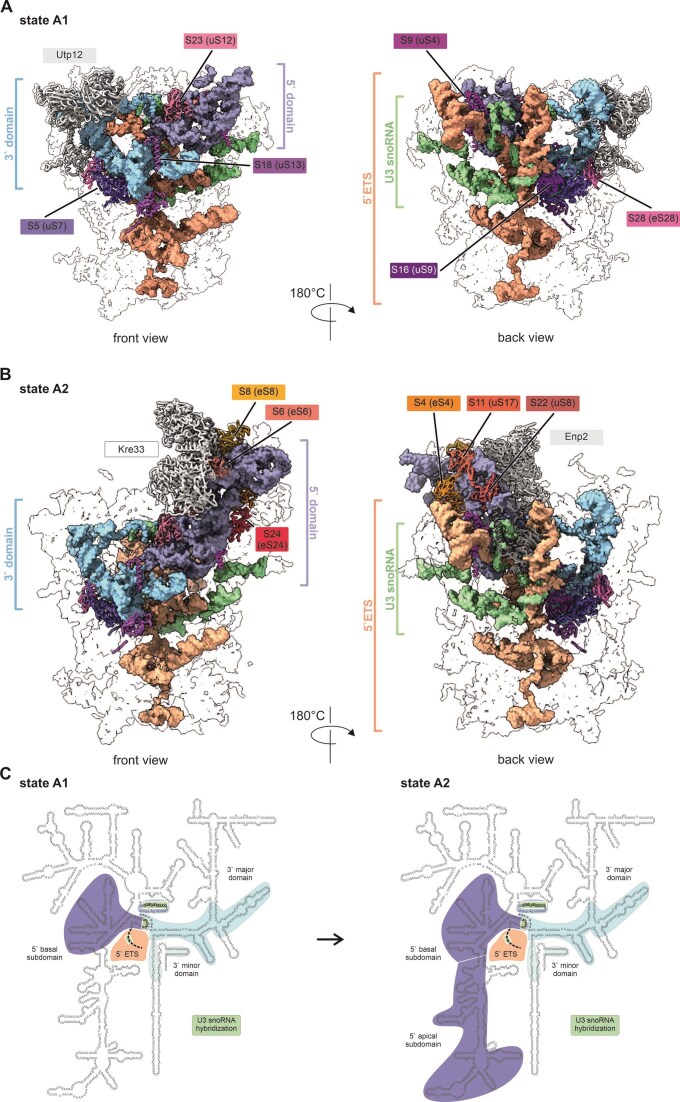
Very early 90S states suggest that domains are compacted in a non-linear manner. The 90S-specific population of the Noc2-TAP particle after depletion of Rlp7 showed three distinct states (see also Supplementary Figs S4B and S7A), all three with a global resolution of about 3.3 Å (Supplementary Fig. S6). (**A**) State A1 only has the 3` and 5` basal subdomains compacted and 6 r-proteins bound as labeled. (**B**) In state A2 the majority of the 5` domain is folded leading to binding of six additional r-proteins as labeled. The 6 r-proteins that are already present at state A1 are colored in the same way but not labeled. (**C**) Schematic representation of the 18S rRNA domain regions that are visible in state A1 and A2. In state A1 mainly the 5` and 3` basal subdomains are structured, while the 5` apical domain is only compacted in state A2. The level of compaction of the 3` domain is the same for state A1 and A2. For the central domain only nucleotides in close proximity to 3` domain that are base pairing with the U3 snoRNA are structured in state A1 and A2. The structure of state A3 closely represents state B observed by the Ye lab [20] and is shown in (Supplementary Fig. S7).

In addition to the early pre-LSU particle, we identified SSU processomes prior to A0 and A1 cleavage in the Rlp7-depleted dataset. After consecutive 3D variability analysis and classification, we could separate three different SSU processome states, named states A1 to A3, each with a global resolution of 3.3Å (Fig. 3 and Supplementary Figs S4B, S6, and S7A). The core particle (state A1) comprised the 5` ETS, the 5` and 3` basal subdomains of the 18S rRNA (Fig. 3C), associated proteins (e.g. UTP-A and UTP-B) and the U3 snoRNP (Supplementary Table S8). Clear structural additions to this core particle are found in states A2 and A3. Our most matured state (termed A3) closely resembled published SSU processome particles, characterized by a fully assembled Kre33 module, a more extensively structured 5` domain, as well as an incorporated and thus well-resolved central domain, but state A3 lacked the UTP-C module (Supplementary Fig. S7C and D, Supplementary Table S8).

### Initial SSU processome core particle

The 5` ETS together with the bound proteins formed a cup-like structure acting as a scaffold for shaping the 3` basal and the 5` basal subdomains seen in state A1 (Fig. 3A and C). From the 3` minor domain, only the lower part of h44 of the 18S rRNA was visible due to interactions with proteins, notably Utp12 and Rps5 (uS7). Utp12 thereby adopts a fully closed conformation, which is only possible when h44 is already established. As this helix is close to the 3` end of the 18S rRNA, this means that the A1 state can only form when the 18S rRNA is already fully transcribed. The central domain was not in its stably folded form and presumably still in a flexible outward rotated state, as we could not resolve it in this structure (discussed below). Only h27 of the central domain was visible, most likely because it was hybridizing with the U3 snoRNA, as it was also observed at other sites (18S rRNA 2–14 nt and 5`ETS) (Supplementary Fig. S7B). The correct positioning of the U3 snoRNA might thus not only prevent the formation of the central pseudoknot but also establish the general topology of the 18S rRNA during the initial phase of assembly and thus pave the way for the structuring of the 3` major domain. The hybrid formation with the 5` end of the 3` major domain, thereby captured the 18S rRNA to direct it towards the central part of the 5` ETS vessel.

Notably, only six r-proteins can be assigned in state A1, five of them universal r-proteins: Rps5 (uS7), Rps9 (uS4), Rps16 (uS9), Rps18 (uS13), Rps23 (uS12), and Rps28 (eS28). As these proteins bind very early to the still unstructured RNA scaffold, they will contribute to the correct folding and assembly of the individual rRNA domains. Rps9 (uS4) and Rps23 (uS12) were thereby the only r-proteins visible in our structure bound to the 5` basal subdomain of the 18S rRNA. Rps9 (uS4) clamps the base of h15, h16, h17, and h18 of the 18S rRNA (approximately nt 435 to 600). It may thus act as a central organizer of the 5` basal subdomain of the 18S rRNA, similarly as it does in the 16S rRNA of bacteria [51]. Rps23 (uS12) binds to a double-stranded region formed by the anterior part of the 5` apical domain (nt 25 to 28) and nt 598 to 601 of the 18S rRNA, and also binds to the tip and base of h18 of the 18S rRNA, thereby organizing the A site.

### Assembly of the 18S rRNA 5`domain

The second structure in our Rlp7-depleted dataset, designated state A2, was identical to state A1 in the lower part of the SSU processome but showed additional structuring of the 5` apical subdomain of the 18S rRNA. This seemed to be connected to binding of six more r-proteins: Rps11 (uS17), Rps6 (eS6), Rps4 (eS4), Rps8 (eS8), Rps24 (eS24) and Rps22 (uS8) as well as the Kre33-complex binding (Fig. 3B and C; Supplementary Table S8). As also found in later particles, Kre33 was present in two copies, with one showing significantly less density than the other protomer. The better defined Kre33 copy thereby clamped together the tips of h14 and h8 of the 18S rRNA. Rps6 (eS6) acts as central organizer of the 5` apical subdomain as it interacts with h6, h8, h10, h13, and with ES3B of the 18S rRNA, as well as Utp20, Enp2, and Kre33.

### Assembly of the 18S rRNA central domain

Similar to state A1, the central domain in state A2 is still flexible and almost not visible in the original reconstruction. Local refinements allowed us to visualize parts of the central domain (Supplementary Fig. S7B). In this conformation, the central domain is not yet closely attached to the core of the particle as in later stages but raised upwards and rotated outwards. This outward position strongly resembles central domain conformations in previous studies [20, 21, 22, 26]. In addition to Utp20, Enp2, and Kre33, the *N*-terminal helices of Utp7 are visible in state A2. Interaction of Utp7 with the N-terminal domain of the now also visible Krr1 might be important for full incorporation of the central domain that we only observe in our state A3. Rps14 (uS11) and Krr1 sit between the base of the central domain and the 5`ETS and might stabilize the central domain both in the outward position (as it is better visible in state A2, where Krr1 is present) and later in the fully incorporated conformation (state A3). Utp13 is located at the opposite side of the central domain and is in contact with Krr1, Rps14 (uS11), and Rrp5. Once the 5` domain is fully structured (as in our state A2), the C-terminal helix (residues 271 to 305) of Krr1 interacts with h11 of the 18S rRNA 5` domain. Krr1 thus spans from the 5` domain to Utp7 at the opposite side of the SSU processome that interacts with its *N*-terminal domain, which gets fully visible at state A3. The globular middle KH domain of Krr1 interacts with Rps14 (uS11), a single-stranded region shortly before h26`, and with the stem and loop of h23, thereby structuring the core of the central domain. This might generate the binding site for Rps1 (eS1) and promote its incorporation. As a consequence, the Utp13 N-domain, which still adopts a relaxed conformation in the state A2 structure, now claps into the fully closed conformation also present in later SSU processome structures. The region from nucleotide 1179–1454 (the peak subdomain) from the 3` major domain is still flexible and not resolved in our structures due to the lack of Enp1. In conclusion, our state A3 structure closely resembles already published states, with an incorporated central domain that is bound by the r-proteins Rps14 (uS11), Rps7 (eS7), Rps13 (uS15), and Rps1 (eS1) (Supplementary Fig. S7C and D).

### Incorporation line of early binding ribosomal proteins into the SSU and LSU

A known limitation of cryo-EM is that flexible, partially assembled domains are hardly depictable and not accessible by this analysis. Thus, inferring a detailed chronological order for assembly factors or r-proteins is not possible. To overcome this limitation, we used an unbiased approach to temporally dissect the binding of early r-proteins to pre-ribosomal particles by SILAC analysis. As it was shown previously that yeast cells preferentially incorporate externally added lysine into freshly synthesized proteins [52], we grew our strain to exponential phase, harvested the cells and removed the growth medium, and transferred them to medium containing ^13^C^15^N-lysine for either 3 or 15 min (Supplementary Fig. S8 and methods section).

Subsequently, we purified very early, co-transcriptionally assembled pre-ribosomal particles (SSU and LSU) using Utp7-TAP as bait protein. While the 5` ETS is transcribed, Utp7 joins the nascent rRNA together with the U3 snoRNA and the UTP-B complex prior to any ribosomal protein [9, 53]. Moreover, Utp7 leaves the pre-ribosome shortly after A0 cleavage together with the U3 snoRNA [54]. The isolated particles were analyzed by western and northern blot (Supplementary Fig. S9) and by qMS for the content of ^13^C^15^N-lysine (heavy lysine) labeled peptides (Supplementary Excel Sheet 1). Utp7-TAP particle composition, given by the total abundances relative to the bait, confirmed homogenous particles with only minimal deviation between the individual experiments and the three biological replicates (Supplementary Table S9).

**Figure 4.**
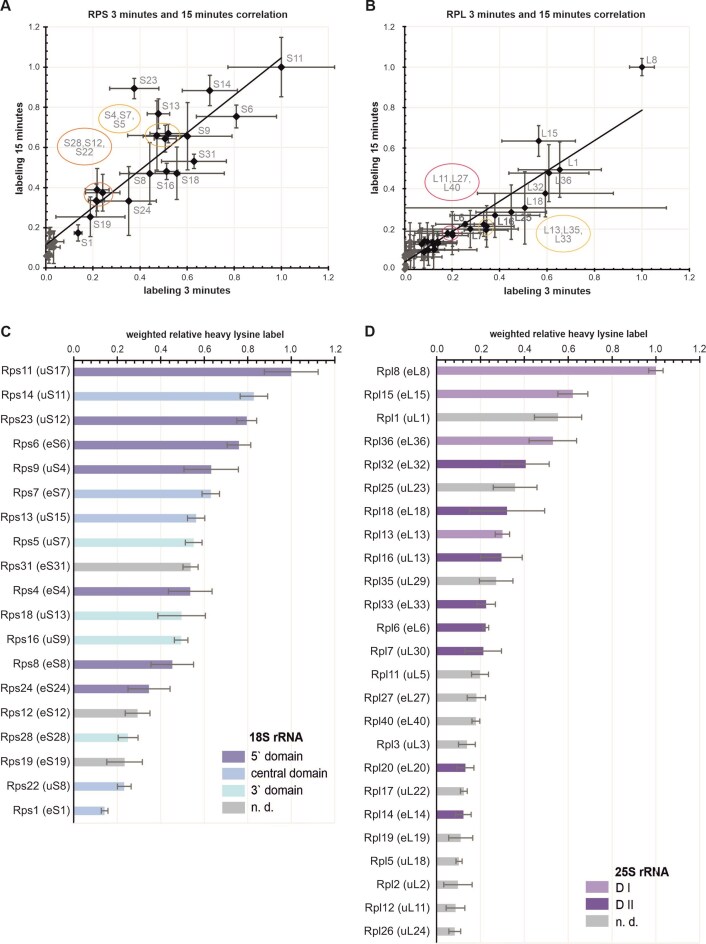
SILAC-labeled ribosomal proteins detected by qMS in samples purified via Utp7-TAP. Strains were incubated in medium containing heavy lysine for 3 or 15 min (Supplementary Fig. S8A). After harvesting, co-transcriptionally assembled pre-ribosomes were isolated using Utp7-TAP as a bait (Supplementary Fig. S9). Three replicates were analyzed by qMS, and the signal intensities of peptides containing ^13^C^15^N-lysine labeled lysine was related to the total peptide intensity (see material and methods, Supplementary Table S9–S13). (**A**) & (**B**) Relative labeling rates of individual r-proteins were obtained by normalization to the highest labeled r-protein for both datasets (3 and 15 min). R-proteins with abundancies below the threshold ratio of <0.1 relative to the bait protein and a relative label of ∼0 for one or two of the six determined values are displayed as gray diamonds and are omitted from Figs 4C and D. A correlation plot of normalized labeling rates for the r-proteins of the small subunit (**A**) shows a Pearson correlation of 0.92, as well as for the r-proteins of the large subunit (**B**), with a Pearson correlation of 0.94. (**C**) & (**D**) Weighted mean and error values of both datasets (see methods section). (**C**) Relative label for the r-proteins of the small subunit, colored by the individual domains of the 18S rRNA to which they bind in our state A3 structure. r-proteins not visible in our structure are represented by gray bars (n.d.). D) Relative label for the r-proteins of the large subunit, colored by the two domains of the 25S rRNA as seen in the very early, cotranscriptional Noc1-Noc2 containing particle [15]. Other r-proteins are shown as gray bars.

Finally, we calculated the ratio of the measured intensities for ^13^C^15^N-lysine containing peptides to total peptide intensity for the individual proteins as determined by qMS (Fig. 4, Supplementary Table S10–S13, Supplementary Fig. S8B, and methods section). The obtained ratios show that proteins known to be incorporated late into the pre-ribosomal particle (e.g. Rps15 (uS19), Rps2 (uS5), Rps3 (uS3)) show no or only very low label after both a labeling period of 3 and 15 min (Supplementary Tables S10 and 11). In addition, peptides derived from assembly factors contained hardly any incorporated ^13^C^15^N-lysine within the short label period (Supplementary Fig. S10). This is consistent with the expected low de novo synthesis rate of assembly factors, because they are recycled during the maturation processes. Therefore, assembly factors have essentially no Pearson correlation between the relative heavy lysine label and the abundance of the bait (∼0.1), while r-proteins of both subunits show a weak positive Pearson correlation (∼0.6) (Supplementary Fig. S10). We observed no stringent correlation with the number of lysine residues in the different ribosomal proteins, which is also obvious by the fact that Rps1 (eS1), which contains the largest number of lysine residues (*n* = 33) of our SSU ribosomal protein dataset, is among the least labeled proteins.

As expected, the relatively heavy lysine label of the individual proteins is lower after 3 min of incubation than in the 15-min samples. Nevertheless, the progression of the labeling ratio of the r-proteins at the two time points followed a similar order. The data showed a Pearson correlation of about 0.92 and 0.94 for the SSU and LSU r-proteins, respectively (Fig. 4A and B). Therefore, we normalized each dataset by the r-proteins with the highest label at both time points (Rps11 (uS17) for the SSU and Rpl8 (eL8) for the LSU) to merge the data and thus enhance the statistical power of the combined results. Of note, only the Rps23 (uS12) data are off the regression line, since this protein shows low labeling after 3 min but pronounced labeling after 15 min of incubation in the presence of ^13^C^15^N-lysine in all three biological replicates. We speculate that the delayed appearance of Rps23 (uS12) at the SSU processome is caused by the complex posttranslational 3,4-dihydroxylation of the highly conserved Pro62 residue [55].

Finally, we determined the order of incorporation of the ribosomal proteins on the basis of the weighted mean values of the normalized relative heavy lysine labels of both timepoints (Fig. 4C and D). We estimated the binding order of 19 SSU r-proteins, including all 16 r-proteins found in our state A3 cryo-EM map, and 25 LSU r-proteins associated with the emerging 35S pre-rRNA.

**Figure 5.**
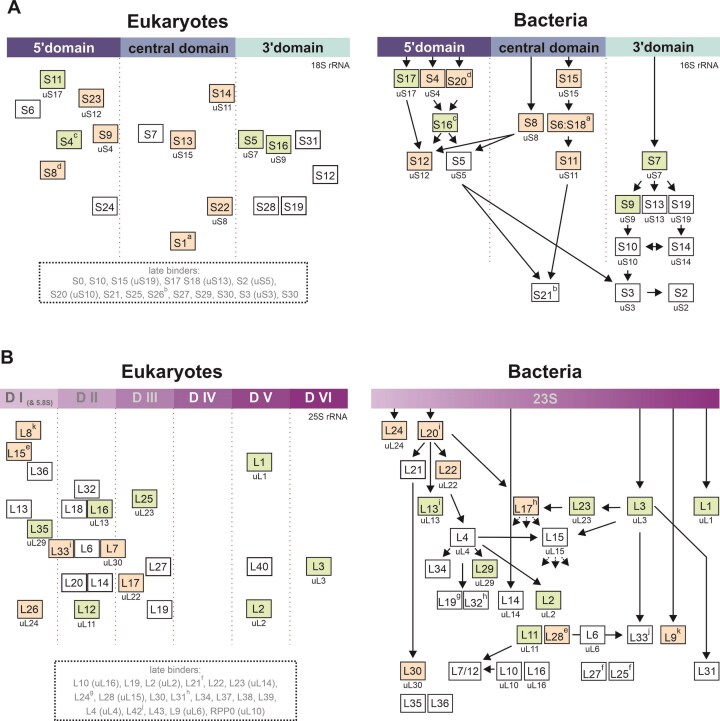
SILAC incorporation order of ribosomal proteins to the rRNA compared to bacteria. The incorporation order of ribosomal proteins of the SSU A) and LSU B), as determined by SILAC labeling (left panel), is compared to the incorporation order of bacteria (right panel) [56, 57]. For the SILAC data, the yeast nomenclature is used, and the ribosomal proteins are assembled according to their rough binding site on the 18S rRNA (**A**) or 25S rRNA (**B**) from early (top) to late (bottom). The universal nomenclature is given below the yeast name. For the assembly line of the bacterial ribosomal proteins, the *Escherichia coli* name is shown together with the universal nomenclature of universally conserved proteins. In small superscript letters (a to k), r-proteins that are not conserved but occupy similar spatial places on the ribosome are marked (Supplementary Table S14). Universally conserved r-proteins, as well as those occupying the same space on the ribosome, are color-coded if their binding time is similar (light green) or different (light orange) from each other.

As shown in Fig. 4C, the earliest incorporated small subunit r-protein is Rps11 (uS17), which associates with h11 in the 5` domain. It is also a primary binder in the bacterial ribosome (Fig. 5). However, in the next step, Rps23 (uS12) and Rps14 (uS11) bind, two universally conserved r-proteins that are known as late binders in bacteria. Subsequently, Rps6 (eS6) binds to the 5` apical subdomain initially structured by Rps11 (uS17) binding. Thereafter, eight ribosomal proteins bind within a narrow timeframe to different regions of the 18S rRNA. This will sculpt the 5` basal subdomain (through Rps9 (uS4) together with Rps23 (uS12)) and 3` basal subdomain (through Rps5 (uS7), Rps16 (uS9) and Rps18 (uS13)) and further proceed to structure the central domain (Rps7 (eS7) and Rps13 (uS15)) and the 5` apical subdomain (Rps4 (eS4) and Rps8 (eS8)). Finally, Rps24 (eS24), Rps28 (eS28), Rps22 (uS8) and Rps1 (eS1) seem to be incorporated. In addition to the mentioned proteins, we also found a significant label content in Rps31 (eS31), Rps12 (eS12), and Rps19 (eS19) that were not depicted in our cryo-EM structures.

For the r-proteins of the LSU, Rpl8 (eL8) showed higher labeling ratios than all other 25S domain I or domain II-associated r-proteins (Fig. 4D). This is in line with its binding to the 5` end of the 25S rRNA that forms a heteroduplex with the 3` end of the 5.8S (h10 ES4) and with h15 at the beginning of the 25S rRNA (nt 120 to 145), while all other r-proteins bind to later transcribed regions. Rpl8 (eL8), thus, might be incorporated co-transcriptionally, once the first 150 nucleotides of the 25S rRNA are transcribed. Rpl15 (eL15) and Rpl36 (eL36), also associated with domain I, showed significantly higher labeling ratios than the domain II proteins Rpl32 (eL32) and Rpl18 (eL18) (Fig. 4B) and therefore might be incorporated shortly after Rpl8 (eL8). Thereafter, Rpl13 (eL13) might finalize domain I assembly. This suggests that binding of the r-proteins to domain I occurred prior to domain II, although both domains can assemble largely independently (Fig. 2). Subsequently, Rpl16 (uL13), Rpl33 (eL33), Rpl6 (eL6), and Rpl7 (uL30) might further structure domain II, which is finalized by Rpl20 (eL20) and Rpl14 (eL14) binding.

## Discussion

### A fully assembled foot is necessary for the structuring of LSU domain I

Under wild-type conditions, Noc2-TAP co-purifies early to intermediate nucleolar pre-LSU particles. The structures solved resemble known states where 25S rRNA domain I, II, and VI, as well as the foot structur,e are associated with r-proteins and assembly factors (Supplementary Fig. S5A) [18, 32]. Upon depletion of the foot structure protein Rlp7, we find a shift of the Noc2-TAP particle population to earlier forms, which is consistent with the literature (Figs 1, 2, and 3) [43, 44, 45]. Our cryo-EM structures of pre-ribosomes isolated from the depleted strain show that under these conditions, Noc2-TAP copurifies a very early co-transcriptional stage of LSU precursor particles together with three different SSU precursor particles. Since we detected both 35S and 27SA_2_ pre-rRNA in these preparations, it is tempting to speculate that the 27SA_2_ pre-rRNA is present in the LSU precursor, while the SSU processomes contain the 35S pre-rRNA. However, the assembly of both subunits occurs simultaneously, and LSU assembly already starts at the 35S rRNA [58, 59]. Thus, the pre-LSU particles we detected in our cryo-EM analysis could contain either 27SA_2_ or 35S pre-rRNA. In line with this suggestion, Noc2 co-sedimented with the 60S and 90S peaks in sucrose gradient centrifugation (Supplementary Fig. S2C).

Pre-LSU particles purified in the absence of Rlp7 still had domain II of the 25S rRNA readily folded, and the conformation of this domain was almost perfectly superposable with this region in the recently published very early pre-LSU particle purified with an rRNA mimic [15]. In contrast, domain I of the 25S rRNA and the foot region formed by ITS2 and associated factors were not visible in our reconstruction (Fig. 2). Upon Rlp7 depletion, foot factors like Nop7, Erb1, and Ytm1 failed to bind to the pre-ribosome and to form the characteristic foot structure. This prevented compaction of domain I, even though domain I assembly factor Ebp2 was bound to the particle (Fig. 1, Supplementary Fig. S2). Since Rlp7 is mostly restricted to the foot structure, it is unlikely to play a direct role in domain I compaction. More likely, the inability to load other ITS2 binding factors, in particular Erb1, might be the reason for the failure to structure this domain. Erb1 acts as a hub protein that binds at the foot structure with its C-terminal end but meanders through wide parts of the domain I and beyond [18], thereby contacting different assembly factors and r-proteins. It is tempting to speculate that Erb1 is the critical factor to initiate the structuring of this domain by connecting the foot and domain I of the 25S rRNA and possibly by supporting the loading of early joining r-proteins like Rpl15 (eL15) and Rpl36 (eL36).

### Incorporation line of the early joining ribosomal proteins into the LSU

Our SILAC analyses allowed us to determine the co-transcriptional assembly line of the LSU r-proteins to the 25S rRNA (Fig. 4D and Fig. 5B). Rpl8 (eL8) was incorporated first into domain I, where it binds to the very 5` end of the 25S rRNA which forms a heteroduplex with the (previously transcribed) 3` end of the 5.8S rRNA (helix 10). Early incorporation of Rpl8 (eL8) is consistent with data from literature, showing that depletion of Rpl15 (eL15) or Rpl36 (eL36) does not affect Rpl8 (eL8) levels in early pre-ribosomal particles [60]. In contrast, depletion of Rpl8 (eL8) leads to reduced levels of Rpl13 (eL13), Rpl15 (eL15), and Rpl36 (eL36) and of all foot factors on early pre-ribosomes, suggesting that incorporation of Rpl8 (eL8) precedes foot assembly and the incorporation of other large subunit r-proteins [61, 62]. This indicates that Rpl8 (eL8) binding to h10 is crucial to initially determine the ITS2 structure, which then allows recruitment of the foot factor proteins. The foot factors, then, orchestrate the compaction of the domain I as shown by our structural data.

Our SILAC data can further resolve the order of incorporation to Rpl8 (eL8), Rpl15 (eL15), and Rpl36 (eL36), which is consistent with the assembly hierarchy inferred from depleting Rpl8 (eL8), Rpl15 (eL15), and Rpl36 (eL36) [63]. Compared to the previously mentioned domain I-associated ribosomal proteins, Rpl13 (eL13) is incorporated with a significant delay. Since Rpl13 (eL13) binds in the region around nucleotide 78–110, while Rpl36 (eL36) binds in the region of nucleotides 264 to 300, the assembly does not occur in a co-transcriptional manner.

Domain II assembly is likely initiated by co-transcriptional binding of the Noc1/Noc2 complex to helix 2 formed between the 5` end of the 5.8S rRNA and nucleotides 410–419 of the 25S rRNA [15, 63]. The domain II can be separated into two nodes (node I and II) that bridge domain II with domain I and domain VI, respectively [63]. According to our SILAC data, the first incorporated ribosomal protein to domain II is Rpl32 (eL32). Rpl32 (eL32) is a node I protein and might be important to determine the overall structure of the domain and/or affect Mak16 recruitment. This is based on the fact that its depletion interferes with the incorporation of the assembly factor Rrp1, which does show direct contact to that ribosomal protein [63]. Subsequently, Rpl18 (eL18) and Rpl16 (uL13) might be incorporated (Fig. 4D and 5B) and further define the structure of node I and node II, respectively. Thereafter, Rpl32 (eL32), Rpl6 (eL6), and Rpl7 (uL30) bind within a narrow time frame and further sculpt domain II. The node II protein Rpl14 (eL14), which was proposed to act in concert with Rpl16 (uL13) as a clamp for stabilizing the docking of domain II to domain VI, only joins at a later stage shortly after Rpl20 (eL20). No significant label was found in the proximal stem proteins Rpl17 (uL22), Rpl26 (uL24), Rpl35 (uL29), and Rpl37 (eL37) [63], indicating that they are incorporated at a later stage.

Finally, incorporation of Rpl4 (uL4) finalizes domain II compaction as detected in our cryo-EM analysis. These SILAC data are consistent with data from the Woolford and Milkereit labs that showed that Rpl14 (eL14) and Rpl20 (eL20) are diminished on pre-ribosomes after depletion of Rpl7 (uL30) [44, 62].

### Compaction of the 18S domains in the SSU processome is non-linear

The temporal order of 18S rRNA domain assembly in the co-transcriptional SSU processome is still inconclusive, as states where the central domain is incorporated before the 5`apical domain were observed (in *Chaetomium thermophilus* [26]), but also states with a well-structured 5` domain but a still flexible central domain [12, 64].

Our data suggest that incorporation of the 18S rRNA domains follows a complex pattern with the 5` basal subdomain and the anterior part of the 3` major domain being integrated first (state A1, Fig. 3A). This part contains the crucial sites of the small subunit (A and P tRNA binding sites) and is therefore particularly important. The 5`ETS, together with its associated assembly factors and the U3 snoRNA that forms heteroduplexes with 5`ETS and 18S rRNA, with its associated protein,s provide the initial framework for sthe tructuring of the SSU rRNA.

According to the available structural data, the main organizers for the compaction of the central domain are Rps14 (uS11), Krr1, and Utp7. In contrast to our outward-rotated central domain in state A2, in most proposed SSU processome maturation schemes, the central domain is already fully incorporated very early, prior to further assembly events occurring at the platform [26]. However, in the majority of studies, mostly (but not exclusively) states were identified where the central domain as well as the 5`domain and Kre33 module are both already assembled. When we compared our earlier states to published SSU processome intermediates, we found that our state A2 closely resembles the SSU processome particle from Barandun and coworkers [21]. As this state was originally accumulated under starvation conditions, it was interpreted as a temporarily unproductive state that possibly can reenter the maturation pathway upon changed conditions. Conformations with outward central domain were also observed in multiple studies where the particles were not derived from impaired growth conditions, but partially after depletion of factors involved in ribosome biogenesis [11, 20] as in our case. Whether this reflects alternative pathways or states that only accumulate when ribosome biogenesis is blocked or temporarily shut down remains to be resolved. Differences between observed states may also be introduced using different tagged bait proteins for purification, as this can also influence the structuring of individual domains (e.g. Kre33-tagging in close proximity of the 5`apical domain).

### The incorporation line of early binding SSU ribosomal proteins

Based on our SILAC analysis, the incorporation of SSU r-proteins did not follow a strict linear mode from the 5` end towards the 3` end. Instead, we find a few proteins that initiated the assembly process for individual domains or subdomains (Fig. 4C and 5A). These are Rps11 (uS17), Rps23 (uS12), and Rps14 (uS11), which were the first proteins binding to the 5` domain, the 5` basal subdomain, and the central domain, respectively. We propose that these binding events serve to define the individual domains. At our earliest cryo-EM stage, these domains are still flexible, precluding the detection of these proteins. The reason for the early incorporation of these proteins in eukaryotic cells is likely found in the fact that they represent important interaction hubs for the SSU processome. Rps11 (uS17) binding might pave the ground for later incorporation of Rps6 (eS6), which seems to initiate 5` domain compaction by simultaneously interacting with Kre33, Enp2, and Utp20. Rps23 (uS12) interacts with Noc14 and Bms1 in the center of the SSU processome, while Rps14 (uS11) interacts with Krr1 and Utp13 at the base of the SSU processome. Rps6 (eS6) and Rps14 (uS11) are thereby localized at the opposite side of the base and correctly position the 18S rRNA 5` domain and central domain in the context of the SSU processome. Subsequent binding of further r-proteins within a narrow time window might then compact the individual domains. This is somehow reminiscent of the situation in bacteria, where the individual domains are assembled independently and later consolidated [62, 65].

Rps9 (uS4) thereby further structures the 5` basal subdomain, while Rps5 (uS7) initiates structuring of the 3` basal subdomains. Thus, while uS4 is the first r-protein supposed to be incorporated into the bacterial ribosome [66], it might only bind significantly after Rps23 (uS12) in eukaryotes. This suggests that the above-mentioned interaction with Bms1 and Noc4 could act to stabilize Rps23 (uS12) and promote its earlier incorporation. Interestingly, uS12 was recently reported to support stable uS4 incorporation during small subunit assembly in bacteria [67]. This underlines the tight connection of uS4 and uS12. The last co-transcriptionally joining r-proteins Rps24 (eS24; to the 5` domain), Rps1 (eS1; to the central domain) and Rps28 (eS28; to the 3` basal subdomain) seemed to seal the individual domains and link them stably in the context of the SSU processome.

Surprisingly, those proteins showing low labeling ratios and hence binding last to the pre-ribosome are spatially positioned in close proximity to the proteins incorporated first into the pre-ribosome, as indicated by the highest labeling ratios. This holds true for the Rps11 (uS17) / Rps8 (eS8) and Rps4 (eS4) / Rps24 (eS24) pairs in the 5` domain, the Rps14 (uS11) / Rps1 (eS1) and Rps13 (uS15) / Rps22 (uS8) pairs in the central domain and the Rps5 (uS7) / Rps28 (eS28) pair in the 3` domain. This suggests that the first binding proteins define the individual domains, while late binding proteins seal these domains in the context of the SSU processome.

Taken together, very early assembly of the small subunit r-proteins does not follow a strictly linear, co-transcriptional mode, but defines modules that are integrated into the context of the SSU processome.

### External and internal transcribed spacers act as initial scaffolds

From a mechanistic point of view, initial compaction of the 18S rRNA domains is guided by the transacting U3 snoRNA, which temporarily base pairs with the first transcribed element of the initial 35S pre-rRNA, the 5` ETS and with the 18S rRNA to facilitate correct positioning of the rRNA domains in the context of the maturating SSU processome. In addition, the 5` ETS provides binding sites for transacting factors that interact with r-proteins to position them precisely for sculpturing the 18S rRNA. This includes Pwp2, Utp21, Utp12 and Bsm1, all of which also interact with the 5` ETS. Indeed, we were recently able to show that the 5` ETS is strictly required to bind assembly factors and small subunit r-proteins into full-length 18S rRNA [43]. Thus, this only temporarily present spacer segment is required for stably anchoring the maturation factors that keep the 18S rRNA domains in place or direct the r-proteins to their correct destination.

Similar mechanisms seem to be employed during large subunit formation and folding of the large 25S rRNA with its 6 individual domains. In that case, the 5.8S rRNA holds the 25S domains I and II in place, similar to the U3 snoRNA during small subunit formation. Moreover, the hub protein Erb1, which is associated with the ITS2 spacer RNA containing foot structure, coordinates the binding of both transacting assembly factors and r-proteins. As in the case of the 5` ETS in the SSU processome, ITS2 gets degraded by exonucleases once assembly is completed.

Thus, the conserved external and internal transcribed spacers provide binding sites for transacting proteins that crucially drive the initial compaction of the rRNA, explaining their spatial conservation in eukaryotes. Once the r-proteins are incorporated and thus provide stability to the folded core, these ETS or ITS sequences can be removed, and associated assembly factors can be released, which licenses the particle for downstream maturation.

### Comparison of r-protein assembly in eukaryotes and bacteria

Fig. 5 depicts the similarities and differences in the assembly order of early binding ribosomal proteins from eukaryotes (this study) and bacteria [56, 57] for both subunits. While some of the r-proteins associate with the ribosomal subunits in a conserved order (depicted in light green), others do not (depicted in light orange). As mentioned above, this difference could be caused by the interaction of r-proteins with assembly factors that are anchored to ETS or ITS sequences and which stabilize initially labile interaction with the rRNA. Thus, there seems to be a certain flexibility in the initial steps of domain assembly.

Another cause for these differences is phylum-specific r-proteins, some of which show overlapping or similar spatial occupancy on the ribosome but nevertheless bind at different stages: For example, eS6 and eS8 bind to a similar region as the primary binder bS20, but encounter the SSU rRNA from the opposite side. However, both eukaryotic r-proteins bind significantly later than the bacterial protein. Another example is eS1, which is the last r-protein to associate with the central domain of the SSU rRNA and binds overlapping regions with the secondary binders bS6/bS18 (Supplementary Table S14).

In contrast, eS4 and bS16 bind to the same area on the LSU and are both secondary binders (Fig. 5A). These r-proteins might be required to correctly align the 5` domain and central domain of the SSU rRNA and thus be crucial for the global architecture of the small ribosomal subunit. Thus, while there might be some flexibility tolerated in the order of structuring the individual domains in the eukaryotic and bacterial phyla, the global overall compaction and final domain consolidation are likely dictated by the overall highly conserved structure of the ribosome. Interestingly, the order of r-protein binding seems to be more conserved for the 3` domain of the SSU. This could be caused by the fact that this domain contains fewer eukaryotic insertion sequences than the central domain and 5` domain of the SSU rRNA.

From the universal conserved r-proteins of the LSU, uL24 and uL22 are among the first ribosomal proteins to be incorporated into the ribosome of bacteria (Fig. 5B) [56, 57]. These r-proteins, together with uL4, are important for the structuring of the poly peptide exit tunnel (PET). This suggests considerable selection pressure to structure the PET at an early stage of maturation in *E. coli*. In contrast, in the eukaryotic ribosome, these r-proteins are only incorporated at a later stage. One reason for this difference might be that the PET is protected and occupied throughout eukaryotic ribosome biogenesis by helices from assembly factors (reviewed in [68, 69]). Within the nucleus, this task is managed by the C-terminal helix of the assembly factor Nog1. Nog1 joins LSU maturation at an early stage, shortly after the 25S rRNA domain I and domain II are formed [18, 70]. Another difference might be the presence of the 5.8S rRNA that is also important for PET formation [71] and for recruitment of both Rpl8 (eL8) and Noc1/Noc2 that are crucial for 25S rRNA domain I and domain II structuring, respectively. Together with the fact that domain I structuring is orchestrated by ITS1-bound assembly factors, it is not surprising that these early stages of eukaryotic LSU maturation are different from bacteria. Indeed, most of the r-proteins we detected in our SILAC analysis as strongly labeled are associated with the 25S rRNA domain I and domain II. The assembly order of the other domains in the LSU might be more similar in bacteria and eukaryotes, since uL23, uL1, and uL3 show comparably binding behavior in *E. coli* and yeast. This finally leads to the near-complete formation of the solvent accessible side of the LSU.

Taken together, we could resolve the assembly line of early joining eukaryotic r-proteins and gain deep insights into rRNA domain compaction. This involves the identification of individual domains by seed proteins, followed by their independent compaction and domain consolidation. While this overarching principle of ribosome assembly seems evolutionarily conserved, the exact sequence by which rRNAs and r-proteins are assembled has diverged from bacteria to eukaryotes, where it is orchestrated by spacer sequences and assembly factors.

## Supplementary Material

gkag036_Supplemental_Files

## Data Availability

Strains generated in this study are available from the corresponding authors upon request. Source data are provided with this paper. Structural data generated in this study were deposited in the PDB (Rlp7-depleted dataset, LSU particle: 9QJC) and EMDB (accession codes: EMD-53201: Rlp7-depleted dataset, LSU particle; EMD-55356: non-depleted dataset, LSU particle - state 1; EMD-55368: non-depleted dataset, LSU particle - state 2; EMD-55840: non-depleted dataset, LSU particle - state 3; EMD-55843: Rlp7-depleted dataset, SSU particle - state A1; EMD-55860: Rlp7-depleted dataset, SSU particle - state A2; EMD-55874: Rlp7-depleted dataset, SSU particle - state A3). The cryo-EM raw data (unprocessed micrographs) are deposited in the EMPIAR database (accession codes EMPIAR-13049 (non-depleted dataset) and EMPIAR-13048 (Rlp7-depleted dataset)). The mass spectrometry proteomics data have been deposited to the ProteomeXchange Consortium via the PRIDE [72] partner repository with the dataset identifier PXD058994.

## Notes

Present address: Laboratoire de Biologie Structurale de la Cellule (BIOC), UMR 7654-CNRS, École polytechnique, Institut Polytechnique de Paris, 91128 Palaiseau CEDEX, France
