# Supplementary material for: A comprehensive view on r-protein binding and rRNA domain structuring during early eukaryotic ribosome formation
Source: Nucleic Acids Res. 2026 Jan 22;54(3):gkag036. doi: 10.1093/nar/gkag036 (PMC12825303; doi:10.1093/nar/gkag036)
Supplement: gkag036_Supplemental_Files [file gkag036_supplemental_files.zip › SUPPLEMENTS_revised.pdf]

Supplemental Information

A comprehensive view on r- protein binding and rRNA domain structuring during early eukaryotic ribosome formation

Authors

Magdalena Gerhalter[1,7,§], Michael Prattes[1,2,7], Lorenz Emanuel Grundmann[3,6,7], Irina Grishkovskaya[3], Enrico F. Semeraro[1,2,4], Gertrude Zisser[1]; Harald Kotisch[3], Juliane Merl-Pham[5], Stefanie M. Hauck[5], David Haselbach[3,*], and Helmut Bergler[1,2,4,*]

*Correspondence should be addressed to D.H. or H.B.

Affiliations

[1]Institute of Molecular Biosciences, University of Graz, Graz, 8010, Austria

[2]BioTechMed-Graz, Graz, Austria

[3]Research Institute of Molecular Pathology (IMP), Vienna BioCenter, Vienna, 1030, Austria

[4]Field of Excellence BioHealth, University of Graz, Graz, Graz, 8010, Austria

[5]Metabolomics and Proteomics Core, Helmholtz Center Munich, German Center for Environmental Health GmbH, D-80939, Germany

[6]Vienna BioCenter PhD Program, Doctoral School of the University of Vienna and Medical University of Vienna, 1030 Vienna, Austria.

[7]These authors contributed equally

[§]current address: Laboratoire de Biologie Structurale de la Cellule (BIOC), UMR 7654-CNRS, École polytechnique, Institut Polytechnique de Paris, 91128 Palaiseau CEDEX, France

Keywords

Ribosome biogenesis, SSU processome, LSU assembly, rRNA domain folding, ribosomal protein assembly, single particle cryo-EM

Supplementary Tables

Supplementary table 1: *Saccharomyces cerevisiae* strains used in this study.

**Strain**	**genotype**	**reference or source**
Noc2-TAP P_{GAL1}-Rlp7	*Mat a; ura3; trp1; leu2; his3; lys2; P_{GAL1}-HA-RLP7::TRP1; NOC2-TAP::HisMX*	(Gerhalter et al. 2024)
Nop7-TAP P_{GAL1}-Rlp7	*Mat a; ura3; trp1; leu2; his3; lys2; P_{GAL1}-HA-RLP7::TRP1; NOP7-TAP::HisMX*	This study
Bud20-TAP P_{GAL1}-Rlp7	*Mat a; ura3; trp1; leu2; his3; lys2; P_{GAL1}-HA-RLP7::TRP1; BUD20-TAP::HisMX*	This study
Utp7-TAP	*Mat a; ura3; trp1; leu2; his3; Utp7-TAP::HisMX*	This study

Supplementary table 2: Plasmids and primers used for strain generation.

**Plasmid**		**Source**
Tagging plasmid: pFA6a-TAP-hisMX		(Janke et al. 2004)
**Name**	**primer – sequence (5`-3`)**	**Purpose**
Nop7_S2	GACAAAATTTTTGAGAGGCTATTGGAAAAGAAGAGAAAAAT CGATGAATTCGAGCTCG	Tagging PCR
Nop7_F2	AAATTGCCAAACAAAAAGCTAAACTGAATAAACTAGATTCCA AGAAACGGATCCCCGGGTTAATTAA	Tagging PCR
Bud20_S2	AAAAAGTGTATATGCCTATTTTTGGTTCTGATCTTTACTTT ACTGTTAATCGATGAATTCGAGCTCG	Tagging PCR
Bud20_F2	ATACCGCTGCCGTAACAGAGGCAGAGTCTACTGCCTCAG CCAGTACTCGGATCCCCGGGTTAATTAA	Tagging PCR
Utp7_F2	GACCACAAGGATGTCATCGAAGAGGCATTGAGCAGATTCGGCC GGATCCCCGGGTTAATTAA	Tagging PCR
Utp7_S2	ACATACATAATCATTTAAGTTTTTTTTTTAAAGATATTTCGATATC GATGAATTCGAGCTCG	Tagging PCR
Bud20_ctrl_fwd	AATACCGCTGCCGTAAC	cPCR Tagging
Nop7_ctrl_fwd	GCTGATAAGGATGTCAATAAG	cPCR Tagging
Utp7_ctrl_fwd	GAACAAACTGCCAGCAGATAC	cPCR Tagging
TAP-tag_rev	CGGTTGGCTGCTGAGACGGC	cPCR Tagging
All primers listed purchased by Merck / Sigma-Aldrich		

Supplementary table 3: Antibodies used in this study.

**Primary Antibody**		
Name	Dilution	Origin
α-Arx1	1:5 000	M. Fromont-Racine
α-Cbp	1:5.000	*Merck / Sigma - Aldrich*
α-Cic1	1:5 000	University of Stuttgart
α-Ebp2	1:5.000	M.A. McAlear
α-Erb1	1:5 000	J. d. l. Cruz
α-Has1	1:5 000	J. d. l. Cruz
α-Mex67	1:10 000	E. Hurt
α-Nmd3	1:4.000	A. W. Johnson
α-Noc1	1:5.000	P. Milkereit
α-Noc2	1:5 000	P. Milkereit
α-Noc3	1:5.000	P. Milkereit
α-Nog1	1:5.000	M. Fromont-Racine
α-Npa1	1:5.000	Y. Henry
α-Nsa2	1:5.000	M. Fromont-Racine
α-Rok1	1:5 000	K. Karbstein
α-Rpa135	1:3.000	M. Oaks
α-Rlp10	1:2.000	B.L. Trumpower
α-Rpl16	1:40.000	S. Rospert
α-Rps3	1:50:000	M. Seedorf
α-Rps8	1:5.000	G. Dieci
α-Rsa3	1:10.000	Y. Henry
α-Sof1	1:300	E. Hurt
α-Ytm1	1:5.000	J. d. l. Cruz
		All primary antibodies are rabbit antibodies
**Secondary Antibody / conjugated Antibody**		
Name	Dilution	Origin
α-rabbit	1:15.000	*Merck / Sigma - Aldrich*
α-HA	1:5.000	*Roche*

Supplementary table 4: DNA-Oligos used for northern blot; for the binding site of the different probes see Supplementary Figure S1A.

**Northern Blot**		
probe name	Sequence (5`-3`)	Source
DA_2	GACTCTCCATCTCTTGTCTTCTTG	Merck / Sigma Aldrich
A_2A_3	TGTTACCTCTGGGCCC	Merck / Sigma Aldrich
EC_2	GGCCAGCAATTTCAAGTTA	Merck / Sigma Aldrich
U3 snoRNA	GGATTGCGGACCAAGCTAA	Merck / Sigma Aldrich
25S	CTCCGCTTATTGATATGC	Merck / Sigma Aldrich
18S	CATGGCTTAATCTTTGAGAC	Merck / Sigma Aldrich
5.8S	GCGTTCTTCATCGATGC	Merck / Sigma Aldrich

Supplementary table 5: cryoEM table

	Rlp7 depleted sample				undepleted sample
	**state A1**	**state A2**	**state A3**	**60S particle**	**60S particles**
Microscope	Titan Krios G4i				
Voltage [keV]	300				
Camera	Falcon 4				
Magnification	81,000				
Pixel size at detector [Å/px2]	0.7445				
Total electron exposure [e-/Å2]	40				
Collected frames [No.]	40				
Defocus range [µm]	-2.0 to -4.0				
Energy filter slit width [eV]	2				
Automation software	Thermo Fisher EPU				
Micrographs collected [No.]	11,250				10,272
Total extracted particles [No.]	504,521				679,870
Final particle images [No.]	9,474	8,299	5,482	28,095	15,424
Resolution global [Å]	3.2	3.7	3.4	3.2	2.9
FSC-threshold	0.143				
Resolution range [Å]	3.4 - 12.5	3.4 - 12.8	3.3 - 11.8	2.7 - 8.3	3.0-8.7
Point-group symmetry	C1				
Plunging	Leica GP1 autofunction				
Grid type	3.5/1 prefloated 2 nm carbon				
Adsorption time	30 s				
Temperature and humidity	4 °C, 75 %				
Blotting time	2 s				
Volume	4 µl				
Plotting	Frontside				

Supplementary table 6: Composition of the premature 60S particle isolated via Noc2-TAP from the Rlp7 depleted dataset.

**RNAs**	**Description**		**60S particles Rlp7 depl.**
25S	25S ribosomal RNA		+
5.8S	5.8S ribosomal RNA		+
ITS2	Internal Transcribed Spacer 2		-
**proteins**	**Protein names**	**UniProt**	**Rlp7 depl.**
Rpl14 (eL14)	Large ribosomal subunit protein eL14A	RL14A_YEAST	+
Rpl16 (uL13)	Large ribosomal subunit protein uL13A	RL16A_YEAST	+
Rpl18 (eL18)	Large ribosomal subunit protein eL18A	RL18A_YEAST	+
Rpl20 (eL20)	Large ribosomal subunit protein eL20A	RL20A_YEAST	+
Rpl32 (eL32)	Large ribosomal subunit protein eL32	RL32_YEAST	+
Rpl33 (eL33)	Large ribosomal subunit protein eL33A	RL33A_YEAST	+
Rpl4 (uL4)	Large ribosomal subunit protein uL4A	RL4A_YEAST	+
Rpl7 (uL30)	Large ribosomal subunit protein uL30A	RL7A_YEAST	+
Rpl6 (eL6)	Large ribosomal subunit protein eL6A	RL6A_YEAST	+
Noc2	Nucleolar complex protein 2	NOC2_YEAST	+
Rrp1	Ribosomal RNA-processing protein 1	RRP1_YEAST	+
Noc1/Mak21	Ribosome biogenesis protein MAK21	MAK21_YEAST	+
Mak16	Protein MAK16	MAK16_YEAST	+
Nop4	Nucleolar protein 4	NOP4_YEAST	+

Supplementary table 7: Model Statistic Noc2-TAP Rlp7-depleted 60S moiety

**PDB-ID**	PDB-9QJC
**Initial Model used**	PDB-8E5T, AF2
**Model-resolution**	3.3
**FSC threshold**	0.5
**Map sharpening B-factor [Åˆ2]**	67.8
**Model composition**	
**Non-hydrogen atoms**	34 836
**Protein residues**	2 970
**Nucleotides**	526
**B factors [Å^2]**	
**Protein**	27.63/203.90/83.88
**Nucleotides**	22.92/292.07/109.16
**R.m.s deviations**	
**Bond length [Å]**	0.004
**Bond angle [°]**	0.567
**Validation**	
**MolProbability Score**	1.51
**Clashscore**	4.94
**Poor rotamers [%]**	0.44
**Ramachandran Plot**	
**Favored [%]**	96.27
**Allowed [%]**	3.73
**Disallowed [%]**	0

Supplementary table 8: Composition of the premature 90S particles isolated via Noc2-TAP from the Rlp7 depleted dataset.

			90S particles		
**RNAs**	**Description**		**1**	**2**	**3**
18S	18S ribosomal RNA		+	+	+
U3	U3 snoRNA		+	+	+
5`ETS	5` External Transcribed Spacer		+	+	+
**proteins**	**Protein names**	**UniProt**	**1***	**2***	**3#**
Bms1	Ribosome biogenesis protein BMS1	BMS1_YEAST	+	+	+
Emg1/Nep1	Ribosomal RNA small subunit methyltransferase NEP1	NEP1_YEAST	+	+	+
Fcf2	rRNA-processing protein FCF2	FCF2_YEAST	+	+	+
Imp3	U3 small nucleolar ribonucleoprotein protein IMP3	IMP3_YEAST	+	+	+
Imp4	U3 small nucleolar ribonucleoprotein protein IMP4	IMP4_YEAST	+	+	+
Mpp10	U3 small nucleolar RNA-associated protein MPP10	MPP10_YEAST	+	+	+
Noc4	Nucleolar complex protein 4	NOC4_YEAST	+	+	+
Nop1	rRNA 2'-O-methyltransferase fibrillarin	FBRL_YEAST	+	+	+
Nop14	Nucleolar complex protein 14	NOP14_YEAST	+	+	+
Nop56	Nucleolar protein 56	NOP56_YEAST	+	+	+
Nop58	Nucleolar protein 58	NOP58_YEAST	+	+	+
Pno1	Pre-rRNA-processing protein PNO1	PNO1_YEAST	+	+	+
Rcl1	RNA 3'-terminal phosphate cyclase-like protein	RCL1_YEAST	+	+	+
Rrp9	Ribosomal RNA-processing protein 9	RRP9_YEAST	+	+	+
Sas10	Something about silencing protein 10	SAS10_YEAST	+	+	+
Snu13	13 kDa ribonucleoprotein-associated protein	SNU13_YEAST	+	+	+
Sof1	Protein SOF1	DCA13_YEAST	+	+	+
Utp1/Pwp2	Periodic tryptophan protein 2	PWP2_YEAST	+	+	+
Utp10	U3 small nucleolar RNA-associated protein 10	UTP10_YEAST	+	+	+
Utp11	U3 small nucleolar RNA-associated protein 11	UTP11_YEAST	+	+	+
Utp12/Dip2	U3 small nucleolar RNA-associated protein 12	UTP12_YEAST	+	+	+
Utp13	U3 small nucleolar RNA-associated protein 13	UTP13_YEAST	+	+	+
Utp15	U3 small nucleolar RNA-associated protein 15	UTP15_YEAST	+	+	+
Utp16/Bud21	U3 small nucleolar RNA-associated protein 16	UTP16_YEAST	+	+	+
Utp17/Nan1	NET1-associated nuclear protein 1	UTP17_YEAST	+	+	+
Utp18	U3 small nucleolar RNA-associated protein 18	UTP18_YEAST	+	+	+
Utp21	U3 small nucleolar RNA-associated protein 21	UTP21_YEAST	+	+	+
Utp24/Fcf1	rRNA-processing protein FCF1	FCF1_YEAST	+	+	+
Utp30	Ribosome biogenesis protein UTP30	RL1D1_YEAST	+	+	+
Utp4	U3 small nucleolar RNA-associated protein 4	UTP4_YEAST	+	+	+
Utp5	U3 small nucleolar RNA-associated protein 5	UTP5_YEAST	+	+	+
Utp6	U3 small nucleolar RNA-associated protein 6	UTP6_YEAST	+	+	+
Utp7	U3 small nucleolar RNA-associated protein 7	UTP7_YEAST	+	+	+
Utp8	U3 small nucleolar RNA-associated protein 8	UTP8_YEAST	+	+	+
Utp9	U3 small nucleolar RNA-associated protein 9	UTP9_YEAST	+	+	+
Rps16 (uS9)	Small ribosomal subunit protein uS9A	RS16A_YEAST	+	+	+
Rps18 (uS13)	Small ribosomal subunit protein uS13A	RS18A_YEAST	+	+	+
Rps23 (uS12)	Small ribosomal subunit protein uS12A	RS23A_YEAST	+	+	+
Rps28 (eS28)	Small ribosomal subunit protein eS28A	RS28A_YEAST	+	+	+
Rps5 (uS7)	Small ribosomal subunit protein uS7	RS5_YEAST	+	+	+
Rps9 (uS4)	Small ribosomal subunit protein uS4A	RS9A_YEAST	+	+	+
Enp2	Ribosome biogenesis protein ENP2	NOL10_YEAST	-	+	+
Faf1	Protein FAF1	FAF1_YEAST	-	+	+
Kre33	RNA cytidine acetyltransferase	NAT10_YEAST	-	+	+
Lcp5	U3 small nucleolar ribonucleoprotein protein LCP5	LCP5_YEAST	-	+	+
Rrt14	Regulator of rDNA transcription protein 14	RRT14_YEAST	-	+	+
Utp14	U3 small nucleolar RNA-associated protein 14	UTP14_YEAST	-	+	+
Rps11 (uS17)	Small ribosomal subunit protein uS17A	RS11A_YEAST	-	+	+
Rps22 (uS8)	Small ribosomal subunit protein uS8A	RS22A_YEAST	-	+	+
Rps24 (eS24)	Small ribosomal subunit protein eS24A	RS24A_YEAST	-	+	+
Rps4 (eS4)	Small ribosomal subunit protein eS4A	RS4A_YEAST	-	+	+

*: based on 5WLC; #: 6LQQ with UTP30, Rps18 and 5`ETS from 5WLC

Supplementary table 8 (continued): Composition of the premature 90S particles isolated via Noc2-TAP from the Rlp7 depleted dataset.

proteins	Protein names	UniProt	1*	2*	3#
Rps6 (eS6)	Small ribosomal subunit protein eS6A	RS6A_YEAST	-	+	+
Rps8 (eS8)	Small ribosomal subunit protein eS8A	RS8A_YEAST	-	+	+
Krr1	KRR1 small subunit processome component	KRR1_YEAST	-	-	+
Bfr2	Protein BFR2	BFR2_YEAST	-	-	+
Utp20	U3 small nucleolar RNA-associated protein 20	UTP20_YEAST	-	-	+
Rps1 (eS1)	Small ribosomal subunit protein eS1A	RS3A1_YEAST	-	-	+
Rps13 (uS15)	Small ribosomal subunit protein uS15	RS13_YEAST	-	-	+
Rps14 (uS11)	Small ribosomal subunit protein uS11A	RS14A_YEAST	-	-	+
Rps7 (eS7)	Small ribosomal subunit protein eS7B	RS7B_YEAST	-	-	+

*: based on 5WLC; #: based on 6LQQ with UTP30, Rps18 and 5`ETS from 5WLC

Supplementary table 9: Quality control of the qMS measurements. The relative to the bait abundances (>0.1) of individual proteins was calculated and the mean and deviation [% std] within one experiment (3 and 15 minutes labeling) as well as within the three experiments was calculated and is depicted here. Green shades show standard deviation < 15%, while everything >15% is labelled in blue shades.

relative abundance to the bait Utp7								
all experiments			exp. 1		exp. 2		exp. 3	
Protein	mean	% std	mean	% std	mean	% std	mean	% std
RRP5	3.38	8.4	3.31	8.4	3.19	2.2	3.63	6.4
UTP10	2.90	5.7	2.86	2.3	3.09	1.5	2.75	4.1
NOP56	2.49	10.2	2.33	0.5	2.84	1.5	2.31	5.2
NOP1	2.49	8.3	2.30	0.3	2.74	4.7	2.43	3.7
NOP58	1.74	13.8	1.66	1.5	2.04	6.7	1.51	2.1
UTP21	1.38	2.6	1.35	1.1	1.41	2.5	1.38	1.6
UTP4	1.37	2.6	1.40	2.1	1.35	0.7	1.37	2.9
PWP2	1.30	8.8	1.23	0.3	1.46	3.3	1.21	0.0
NAN1	1.27	6.1	1.24	1.9	1.21	1.3	1.36	4.4
MPP10	1.22	13.5	1.07	2.8	1.40	2.4	1.19	12.8
UTP13	1.13	9.7	1.06	1.5	1.27	2.6	1.07	7.9
SOF1	1.11	6.0	1.12	1.1	1.03	1.0	1.18	2.4
UTP22	1.04	9.1	1.06	12.7	1.02	6.6	1.03	5.6
UTP9	1.00	14.8	0.86	2.3	1.16	5.3	1.00	12.8
**UTP7**	**1.00**	**0.0**	**1.00**	**0.0**	**1.00**	**0.0**	**1.00**	**0.0**
Kre33	0.97	24.1	0.87	18.5	1.26	6.9	0.79	8.9
RRP9	0.94	2.3	0.95	2.2	0.93	1.9	0.93	0.9
BMS1	0.92	12.2	0.98	12.3	0.94	9.1	0.85	9.7
UTP6	0.91	3.1	0.88	2.5	0.91	0.8	0.93	2.6
UTP8	0.89	8.6	0.85	1.8	0.84	2.0	0.98	6.9
UTP20	0.87	18.2	0.91	20.2	0.77	18.1	0.93	8.4
UTP15	0.84	6.1	0.84	4.2	0.90	0.5	0.78	0.4
UTP5	0.71	10.1	0.65	0.8	0.69	1.0	0.78	10.5
HAS1	0.69	6.0	0.66	2.0	0.73	4.2	0.67	3.9
CBF5	0.66	26.9	0.47	0.3	0.90	4.0	0.62	2.2
RPS4	0.63	18.6	0.68	4.7	0.75	1.0	0.47	1.9
UTP14	0.63	21.7	0.65	25.9	0.68	15.2	0.56	15.6
IMP4	0.60	13.1	0.55	2.0	0.58	2.7	0.67	15.0
HCA4	0.59	22.6	0.49	9.1	0.77	7.4	0.52	3.2
UTP11	0.55	3.7	0.54	0.6	0.56	0.2	0.56	5.9
IMP3	0.52	5.7	0.50	1.3	0.51	2.6	0.54	6.9
UTP18	0.51	5.3	0.51	2.6	0.53	0.1	0.48	4.9
RPL8	0.50	9.0	0.54	2.2	0.50	7.0	0.47	10.2
KRI1	0.48	28.6	0.37	7.9	0.67	9.7	0.42	1.4
KRR1	0.48	10.3	0.45	3.4	0.48	0.9	0.50	15.0
ESF1	0.48	39.9	0.29	3.8	0.73	2.6	0.40	7.3
RPS11	0.46	25.3	0.59	3.3	0.48	4.0	0.32	16.1

NOP12	0.46	17.9	0.39	3.0	0.56	10.0	0.42	1.7
RPS7	0.45	11.7	0.40	11.2	0.50	2.1	0.45	6.6
BFR2	0.45	27.1	0.30	15.5	0.55	0.6	0.48	18.8
ENP2	0.43	23.5	0.33	14.2	0.55	3.4	0.40	11.3
MRD1	0.43	16.9	0.34	5.5	0.49	5.7	0.45	9.5
RPS5	0.42	13.7	0.43	16.4	0.43	4.7	0.39	14.4
RPS1	0.40	16.1	0.46	7.8	0.43	2.5	0.32	4.3
FCF2	0.39	15.0	0.32	15.9	0.45	0.1	0.39	1.2
RRP12	0.38	16.0	0.41	19.9	0.36	11.0	0.37	9.2
RPS14	0.36	11.0	0.36	2.0	0.41	5.5	0.32	3.1
RPL3	0.36	14.9	0.40	5.4	0.39	1.7	0.29	8.9
NSR1	0.33	27.2	0.23	7.5	0.43	14.3	0.34	10.6
NOP14	0.33	18.3	0.36	19.1	0.29	13.9	0.33	13.8
RPL13	0.32	16.0	0.33	3.8	0.36	2.1	0.25	11.2
RCL1	0.31	13.4	0.32	17.1	0.30	5.9	0.32	13.4
RPS6	0.31	9.4	0.31	9.7	0.31	1.5	0.31	13.0
EMG1	0.30	19.0	0.32	20.8	0.27	11.7	0.32	17.8
EBP2	0.30	19.6	0.26	8.2	0.38	2.0	0.27	11.4
ROK1	0.30	16.9	0.26	15.0	0.34	4.9	0.29	15.6
RPS9	0.30	26.9	0.37	7.8	0.33	2.6	0.19	2.4
NOP9	0.29	17.4	0.25	8.6	0.34	15.6	0.28	0.7
EFG1	0.29	22.6	0.23	8.2	0.38	5.4	0.26	1.1
ECM16	0.28	13.2	0.28	12.5	0.30	13.8	0.26	6.1
RPS16	0.27	10.1	0.29	8.0	0.28	3.6	0.24	7.9
RPL18	0.27	19.5	0.32	7.1	0.29	1.2	0.20	6.0
RPS18	0.27	18.7	0.31	8.3	0.29	0.8	0.20	2.7
RPS3	0.26	26.6	0.31	1.7	0.31	5.3	0.16	1.6
CIC1	0.26	45.0	0.36	43.6	0.22	3.7	0.20	9.0
RPL7	0.26	18.1	0.31	8.5	0.27	1.6	0.20	11.5
RPL40	0.25	57.8	0.06	3.2	0.42	1.1	0.28	1.4
ESF2	0.25	47.8	0.15	5.6	0.42	0.4	0.19	2.5
RPS13	0.25	18.1	0.29	2.8	0.27	0.3	0.19	0.2
RPS8	0.25	14.8	0.27	5.2	0.26	3.6	0.20	11.6
RPL5	0.24	19.4	0.22	6.9	0.30	4.2	0.20	10.5
GAR1	0.23	21.4	0.18	2.1	0.29	10.4	0.23	1.9
MAK21	0.23	11.7	0.21	9.8	0.25	1.5	0.23	13.7
NHP2	0.22	12.9	0.19	0.7	0.25	9.3	0.23	3.9
LCP5	0.21	30.9	0.14	14.1	0.29	4.1	0.20	15.8
RPS24	0.21	15.6	0.17	10.5	0.24	0.1	0.21	5.7
RPL6	0.21	18.0	0.25	9.5	0.21	2.1	0.16	6.1
RPS23	0.20	27.4	0.19	7.8	0.27	3.1	0.14	6.3
ERB1	0.20	21.7	0.20	18.7	0.22	14.8	0.18	25.3
RPL2	0.19	13.1	0.18	1.9	0.23	3.3	0.17	0.4
NOC2	0.19	13.6	0.21	10.5	0.18	3.2	0.16	8.9
RPL16	0.18	19.8	0.23	6.2	0.15	0.9	0.17	7.3
RPL15	0.18	24.8	0.22	3.5	0.21	1.4	0.12	9.4
RPL10	0.18	8.9	0.19	0.7	0.19	5.5	0.16	1.8

RPL19	0.18	22.1	0.15	3.2	0.24	5.6	0.15	0.4
RPL17	0.18	13.1	0.18	4.1	0.20	2.5	0.15	4.5
NOP6	0.18	28.6	0.12	2.9	0.24	9.1	0.18	6.4
PWP1	0.17	30.8	0.12	15.7	0.23	23.3	0.17	1.8
RPS12	0.17	23.2	0.13	12.9	0.22	0.3	0.16	18.3
RPS22	0.17	15.7	0.16	11.9	0.19	0.9	0.15	15.3
MRT4	0.16	25.0	0.22	12.6	0.13	1.2	0.14	3.6
RPS19	0.16	11.8	0.16	5.4	0.19	4.8	0.15	7.6
RPL26	0.16	6.8	0.15	5.1	0.17	6.9	0.16	5.3
RPL9	0.16	39.4	0.23	6.1	0.18	1.4	0.08	11.0
DBP8	0.16	33.7	0.12	0.9	0.23	4.1	0.12	8.9
RPS2	0.16	16.0	0.19	2.8	0.16	6.5	0.13	5.9
RPP0	0.15	17.8	0.13	0.7	0.18	9.0	0.15	16.5
RRP7	0.15	11.1	0.15	12.2	0.14	4.7	0.16	2.6
RPL25	0.15	14.9	0.15	5.1	0.18	6.4	0.13	8.4
RPS28	0.15	14.4	0.16	12.7	0.16	0.1	0.13	14.9
RPL1	0.15	18.0	0.16	4.1	0.17	4.6	0.11	1.4
UTP23	0.15	17.1	0.14	5.4	0.12	10.5	0.18	1.5
RPL4A	0.14	106.4	0.36	8.7	0.04	7.1	0.03	51.9
NOC4	0.14	19.8	0.14	23.1	0.14	11.9	0.14	21.2
RPL12	0.14	22.9	0.14	3.9	0.17	10.7	0.11	18.4
NOP7	0.14	20.2	0.15	19.7	0.12	8.7	0.14	20.3
ENP1	0.14	31.1	0.15	28.4	0.10	14.8	0.16	25.2
RPL11	0.13	9.8	0.15	5.1	0.13	4.4	0.12	4.4
DHR2	0.13	15.0	0.11	0.3	0.15	6.5	0.14	4.5
NOP4	0.13	28.9	0.13	24.8	0.17	12.3	0.10	25.4
RPS0	0.13	23.9	0.10	3.3	0.17	7.9	0.12	9.7
RPL32	0.13	40.9	0.10	7.2	0.20	0.6	0.09	19.6
BRX1	0.13	13.3	0.15	3.6	0.11	9.4	0.13	8.3
FCF1	0.13	10.4	0.13	7.0	0.11	6.7	0.14	1.7
RPL35	0.13	12.6	0.15	7.0	0.12	0.4	0.11	0.5
RRP36	0.12	44.9	0.05	2.6	0.16	8.5	0.16	4.8
RPL14	0.12	25.6	0.16	6.8	0.12	3.5	0.09	12.3
RPL36	0.12	30.3	0.09	3.2	0.11	20.0	0.17	12.6
RPL20	0.12	20.5	0.15	8.1	0.12	0.2	0.09	1.8
RPS17	0.12	16.2	0.11	9.8	0.10	18.3	0.14	6.2
UTP30	0.11	23.7	0.11	26.0	0.10	11.5	0.13	20.1
RPL27	0.11	25.5	0.11	4.9	0.15	6.1	0.08	1.9
NOP13	0.11	40.8	0.13	12.2	0.15	1.5	0.05	12.9
PNO1	0.10	18.5	0.11	17.7	0.12	12.4	0.09	15.1
MAK5	0.10	77.4	0.21	16.4	0.05	15.1	0.05	41.8
RPL33	0.10	12.0	0.10	7.1	0.12	3.9	0.09	0.4
RPL31	0.10	22.9	0.09	3.1	0.13	7.4	0.08	6.4
NOP10	0.10	41.5	0.05	2.2	0.15	14.9	0.10	5.8
RPL21	0.10	24.3	0.12	3.4	0.10	12.9	0.07	14.0
RPL24	0.10	23.7	0.08	3.2	0.13	4.5	0.08	5.3

Supplementary table 10: Labeling ratios of LSU r-proteins with $^{13}C^{15}N$-lysine. Cells were incubated with $^{13}C^{15}N$-lysine for 3 or 15 minutes, after harvesting the cells co-transcriptional ribosomal proteins were isolated by Utp7-TAP. The resulting samples were analyzed by qMS and the ratio of heavy labeled peptides to unlabeled peptides were calculated for every protein. Further the relative abundance to the bait protein Utp7 was calculated for all proteins. sys.= systematic name (Ban et al. 2014); fold change = change 3 to 15 minutes of labeling with heavy lysine. n=3

Protein		relative protein abundance to bait			relative heavy lysine label in protein						
					3 min labeling time			15 min labeling time			fold change
sys.	yeast	mean	std	std [%]	mean	std	std [%]	mean	std	std [%]	
uL1	RPL1	0.146	0.026	18.0	0.043	0.011	26.8	0.092	0.025	27.7	2.2
uL16	RPL10	0.181	0.016	8.9	0.000	0.001	150.0	0.002	0.003	150.0	4.7
uL5	RPL11	0.134	0.013	9.8	0.013	0.003	20.8	0.033	0.020	59.1	2.6
uL11	RPL12	0.140	0.032	22.9	0.005	0.003	70.9	0.023	0.015	64.3	5.0
eL13	RPL13	0.316	0.050	16.0	0.022	0.003	11.8	0.042	0.011	26.9	1.9
eL14	RPL14	0.122	0.031	25.6	0.008	0.003	42.4	0.023	0.009	40.0	2.9
eL15	RPL15	0.183	0.045	24.8	0.037	0.010	27.5	0.118	0.014	12.0	3.2
uL13	RPL16	0.185	0.037	19.8	0.025	0.012	49.8	0.050	0.020	40.8	2.0
uL22	RPL17	0.179	0.023	13.1	0.005	0.002	33.7	0.026	0.003	13.0	4.9
eL18	RPL18	0.268	0.052	19.5	0.033	0.039	117.8	0.057	0.033	58.7	1.7
eL19	RPL19	0.180	0.040	22.1	0.006	0.004	70.9	0.026	0.017	67.3	4.1
uL2	RPL2	0.193	0.025	13.1	0.007	0.008	126.4	0.018	0.014	78.4	2.7
eL20	RPL20	0.121	0.025	20.5	0.009	0.005	55.6	0.024	0.008	34.6	2.6
eL21	RPL21	0.099	0.024	24.3	0.002	0.002	103.8	0.012	0.012	101.3	7.4
eL22	RPL22	0.037	0.012	33.5	0.000	0.000		0.011	0.017	150.0	
uL14	RPL23	0.081	0.029	35.6	0.005	0.005	92.6	0.012	0.010	88.7	2.2
eL24	RPL24	0.096	0.023	23.7	0.001	0.001	85.8	0.021	0.011	50.9	15.0
uL23	RPL25	0.151	0.022	14.9	0.029	0.010	33.1	0.053	0.025	47.4	1.8
uL24	RPL26	0.161	0.011	6.8	0.005	0.003	47.5	0.016	0.007	44.6	3.0

eL27	RPL27	0.114	0.029	25.5	0.013	0.004	33.0	0.031	0.010	32.2	2.4
uL15	RPL28	0.090	0.018	19.6	0.003	0.002	75.2	0.017	0.007	41.8	6.4
uL3	RPL3	0.361	0.054	14.9	0.009	0.004	41.3	0.026	0.009	36.8	2.8
eL30	RPL30	0.065	0.030	45.4	0.008	0.007	87.0	0.025	0.015	58.8	3.3
eL31	RPL31	0.101	0.023	22.9	0.000	0.000	150.0	0.012	0.002	17.5	61.4
eL32	RPL32	0.129	0.053	40.9	0.039	0.019	48.3	0.070	0.022	30.7	1.8
eL33	RPL33	0.102	0.012	12.0	0.022	0.006	26.9	0.036	0.009	24.3	1.6
eL34	RPL34	0.080	0.021	26.9	0.004	0.006	150.0	0.035	0.007	21.1	8.5
uL29	RPL35	0.126	0.016	12.6	0.023	0.008	33.3	0.040	0.019	48.0	1.8
eL36	RPL36	0.122	0.037	30.3	0.040	0.011	27.8	0.089	0.026	29.5	2.2
eL37	RPL37	0.016	0.004	27.4	0.003	0.004	150.0	0.017	0.015	88.4	6.6
eL38	RPL38	0.050	0.011	22.3	0.000	0.000		0.013	0.002	18.1	
eL39	RPL39	0.019	0.004	19.2	0.000	0.000		0.000	0.000		
uL4	RPL4	0.144	0.153	106.4	0.008	0.012	150.0	0.018	0.019	105.1	2.3
eL40	RPL40	0.252	0.146	57.8	0.012	0.009	76.6	0.033	0.003	9.9	2.8
eL42	RPL42	0.043	0.011	24.7	0.001	0.002	150.0	0.026	0.017	67.9	17.5
eL43	RPL43	0.037	0.016	42.6	0.000	0.001	150.0	0.014	0.011	82.5	35.9
uL18	RPL5	0.242	0.047	19.4	0.008	0.003	33.4	0.019	0.003	14.8	2.2
eL6	RPL6	0.206	0.037	18.0	0.017	0.008	48.7	0.042	0.003	6.1	2.5
uL30	RPL7	0.258	0.047	18.1	0.018	0.013	73.2	0.037	0.017	45.8	2.1
eL8	RPL8	0.503	0.045	9.0	0.065	0.003	5.2	0.186	0.008	4.3	2.9
uL6	RPL9	0.160	0.063	39.4	0.000	0.000	150.0	0.005	0.004	87.2	27.7
uL10	RPP0	0.155	0.028	17.8	0.001	0.001	49.0	0.015	0.005	30.9	10.2
P2B	RPP2	0.077	0.023	29.6	0.000	0.001	150.0	0.013	0.011	86.5	29.9

Supplementary table 11: Labeling ratios of SSU r-proteins with $^{13}C^{15}N$-lysine. Cells were incubated with $^{13}C^{15}N$-lysine for 3 or 15 minutes, after harvesting the cells co-transcriptional ribosomal proteins were isolated by Utp7-TAP. The resulting samples were analyzed by qMS and the ratio of heavy labeled peptides to unlabeled peptides were calculated for every protein. Further the relative abundance to the bait protein Utp7 was calculated for all proteins. sys.= systematic name (Ban et al. 2014); fold change = change 3 to 15 minutes of labeling with heavy lysine. n=3

Protein		relative protein abundance to bait			relative heavy lysine label in protein						
					3 min labeling time			15 min labeling time			fold change
sys.	yeast	mean	std	std [%]	mean	std	std [%]	mean	std	std [%]	
uS2	RPS0	0.131	0.031	23.9	0.000	0.000		0.005	0.007	150.0	
eS1	RPS1	0.403	0.065	16.1	0.013	0.002	11.9	0.036	0.008	23.3	2.9
eS10	RPS10	0.054	0.011	19.5	0.001	0.001	102.2	0.026	0.015	57.3	20.5
uS17	RPS11	0.463	0.117	25.3	0.092	0.021	22.6	0.209	0.031	14.8	2.3
eS12	RPS12	0.170	0.039	23.2	0.022	0.007	30.3	0.078	0.019	24.4	3.5
uS15	RPS13	0.251	0.045	18.1	0.044	0.004	9.9	0.160	0.015	9.6	3.6
uS11	RPS14	0.364	0.040	11.0	0.064	0.011	16.8	0.185	0.016	8.6	2.9
uS19	RPS15	0.022	0.004	16.9	0.000	0.000		0.010	0.015	150.0	
uS9	RPS16	0.270	0.027	10.1	0.047	0.004	9.5	0.100	0.008	8.4	2.1
eS17	RPS17	0.118	0.019	16.2	0.003	0.004	102.0	0.027	0.005	17.6	7.8
uS13	RPS18	0.267	0.050	18.7	0.051	0.018	36.2	0.098	0.027	27.7	1.9
eS19	RPS19	0.164	0.019	11.8	0.017	0.013	77.3	0.053	0.021	38.8	3.1
uS5	RPS2	0.158	0.025	16.0	0.002	0.001	43.2	0.023	0.004	16.8	12.9
uS10	RPS20	0.087	0.021	23.7	0.002	0.002	90.0	0.037	0.006	15.3	20.7
eS21	RPS21	0.021	0.007	35.0	0.000	0.000		0.007	0.010	150.0	
uS8	RPS22	0.166	0.026	15.7	0.020	0.003	15.8	0.070	0.017	24.4	3.6
uS12	RPS23	0.201	0.055	27.4	0.035	0.010	27.7	0.187	0.011	5.6	5.4
eS24	RPS24	0.209	0.033	15.6	0.032	0.011	33.3	0.070	0.036	51.5	2.1
eS25	RPS25	0.060	0.008	13.5	0.001	0.001	116.8	0.013	0.004	34.5	11.3
eS26	RPS26	0.048	0.013	26.2	0.000	0.001	150.0	0.013	0.001	10.1	31.5

eS27	RPS27	0.055	0.018	32.3	0.000	0.000		0.000	0.000		
eS28	RPS28	0.147	0.021	14.4	0.020	0.005	23.2	0.081	0.022	27.2	4.1
uS14	RPS29	0.064	0.011	17.2	0.000	0.000		0.001	0.002	150.0	
uS3	RPS3	0.263	0.070	26.6	0.001	0.001	87.3	0.014	0.009	66.0	11.8
eS30	RPS30	0.021	0.009	43.1	0.003	0.003	82.2	0.022	0.017	78.4	7.1
eS31	RPS31	0.061	0.019	31.1	0.058	0.013	21.9	0.111	0.007	6.7	1.9
eS4	RPS4	0.634	0.118	18.6	0.043	0.011	26.2	0.138	0.036	26.1	3.2
uS7	RPS5	0.419	0.057	13.7	0.047	0.004	9.2	0.134	0.014	10.4	2.9
eS6	RPS6	0.310	0.029	9.4	0.074	0.016	21.0	0.157	0.012	7.5	2.1
eS7	RPS7	0.448	0.052	11.7	0.048	0.007	14.9	0.140	0.010	6.9	2.9
eS8	RPS8	0.246	0.036	14.8	0.041	0.012	28.9	0.098	0.032	32.7	2.4
uS4	RPS9	0.296	0.079	26.9	0.055	0.017	31.3	0.137	0.035	25.6	2.5

Supplementary table 12: Weighted labeling ratios of LSU r-proteins with $^{13}C^{15}N$-lysine shown in Figure 4D. Calculations were performed as described in the methods section. n=3

Protein		labelling ratio with heavy lysine												
		3 min.		15 min.		normalized by RPL8								
						3 min.		15 min.		precision				weighted mean
Sys.	yeast	mean	std	mean	std	mean	std	mean	std	β 3 min	β 15 min	β3 + β15	error	
eL8	RPL8	0.065	0.003	0.186	0.008	1.000	0.052	1.000	0.043	367	547	914	0.033	1.000
eL15	RPL15	0.037	0.010	0.118	0.014	0.564	0.155	0.635	0.076	42	174	215	0.068	0.621
uL1	RPL1	0.043	0.011	0.092	0.025	0.654	0.175	0.493	0.137	33	53	86	0.108	0.554
eL36	RPL36	0.040	0.011	0.089	0.026	0.608	0.169	0.476	0.141	35	51	86	0.108	0.530
eL32	RPL32	0.039	0.019	0.070	0.022	0.593	0.286	0.376	0.116	12	75	87	0.107	0.406
uL23	RPL25	0.029	0.010	0.053	0.025	0.449	0.149	0.283	0.134	45	55	101	0.100	0.358
eL18	RPL18	0.033	0.039	0.057	0.033	0.506	0.597	0.305	0.179	3	31	34	0.171	0.321
eL13	RPL13	0.022	0.003	0.042	0.011	0.333	0.039	0.223	0.060	649	277	926	0.033	0.300
uL13	RPL16	0.025	0.012	0.050	0.020	0.380	0.189	0.267	0.109	28	84	112	0.095	0.296
uL29	RPL35	0.023	0.008	0.040	0.019	0.346	0.115	0.213	0.102	76	95	171	0.076	0.272
eL33	RPL33	0.022	0.006	0.036	0.009	0.343	0.092	0.195	0.047	118	446	564	0.042	0.226
eL6	RPL6	0.017	0.008	0.042	0.003	0.254	0.124	0.224	0.014	65	5438	5503	0.013	0.224
uL30	RPL7	0.018	0.013	0.037	0.017	0.276	0.202	0.200	0.092	24	119	144	0.083	0.213
uL5	RPL11	0.013	0.003	0.033	0.020	0.200	0.042	0.179	0.106	576	89	665	0.039	0.198
eL27	RPL27	0.013	0.004	0.031	0.010	0.202	0.066	0.168	0.054	226	340	567	0.042	0.181
eL40	RPL40	0.012	0.009	0.033	0.003	0.180	0.138	0.179	0.018	52	3161	3214	0.018	0.179
uL3	RPL3	0.009	0.004	0.026	0.009	0.138	0.057	0.138	0.051	306	389	695	0.038	0.138
eL20	RPL20	0.009	0.005	0.024	0.008	0.140	0.078	0.129	0.044	165	505	671	0.039	0.131
uL22	RPL17	0.005	0.002	0.026	0.003	0.083	0.028	0.142	0.018	1273	2955	4229	0.015	0.124
eL14	RPL14	0.008	0.003	0.023	0.009	0.122	0.052	0.122	0.049	373	420	793	0.036	0.122
eL19	RPL19	0.006	0.004	0.026	0.017	0.095	0.068	0.138	0.093	219	116	335	0.055	0.110
uL18	RPL5	0.008	0.003	0.019	0.003	0.126	0.042	0.099	0.015	562	4668	5230	0.014	0.102

uL2	RPL2	0.007	0.008	0.018	0.014	0.100	0.127	0.096	0.075	62	176	239	0.065	0.097
uL11	RPL12	0.005	0.003	0.023	0.015	0.071	0.051	0.125	0.080	392	155	547	0.043	0.086
uL24	RPL26	0.005	0.003	0.016	0.007	0.081	0.038	0.085	0.038	678	691	1369	0.027	0.083

Supplementary table 13: Weighted labeling ratios of SSU r-proteins with $^{13}C^{15}N$-lysine shown in Figure 4C. Calculations were performed as described in the methods section. n=3

Protein		labelling ratio with heavy lysine												
		3 min.		15 min.		normalized by RPS11								
						3 min.		15 min.		precision				weighted mean
Sys.	yeast	mean	std	mean	std	mean	std	mean	std	β 3 min	β 15 min	β3 + β15	error	
uS17	RPS11	0.092	0.021	0.209	0.031	1.000	0.226	1.000	0.148	20	46	65	0.124	1.000
uS11	RPS14	0.064	0.011	0.185	0.016	0.696	0.117	0.883	0.076	73	175	248	0.064	0.828
uS12	RPS23	0.035	0.010	0.187	0.011	0.376	0.104	0.894	0.050	92	395	487	0.045	0.795
eS6	RPS6	0.074	0.016	0.157	0.012	0.809	0.170	0.754	0.057	35	309	343	0.054	0.759
uS4	RPS9	0.055	0.017	0.137	0.035	0.602	0.188	0.656	0.168	28	36	64	0.125	0.632
eS7	RPS7	0.048	0.007	0.140	0.010	0.520	0.078	0.670	0.046	166	463	629	0.040	0.630
uS15	RPS13	0.044	0.004	0.160	0.015	0.478	0.048	0.768	0.074	443	183	625	0.040	0.563
uS7	RPS5	0.047	0.004	0.134	0.014	0.507	0.047	0.644	0.067	459	224	682	0.038	0.552
eS31	RPS31	0.058	0.013	0.111	0.007	0.629	0.138	0.531	0.036	53	790	843	0.034	0.537
eS4	RPS4	0.043	0.011	0.138	0.036	0.471	0.124	0.660	0.172	65	34	99	0.100	0.536
uS13	RPS18	0.051	0.018	0.098	0.027	0.556	0.201	0.471	0.131	25	59	83	0.109	0.496
uS9	RPS16	0.047	0.004	0.100	0.008	0.513	0.049	0.481	0.040	425	612	1036	0.031	0.494
eS8	RPS8	0.041	0.012	0.098	0.032	0.441	0.128	0.470	0.154	61	42	104	0.098	0.453
eS24	RPS24	0.032	0.011	0.070	0.036	0.352	0.117	0.333	0.172	73	34	107	0.097	0.346
eS12	RPS12	0.022	0.007	0.078	0.019	0.242	0.073	0.375	0.092	186	119	305	0.057	0.294
eS28	RPS28	0.020	0.005	0.081	0.022	0.218	0.051	0.389	0.106	391	89	480	0.046	0.250
eS19	RPS19	0.017	0.013	0.053	0.021	0.189	0.146	0.254	0.099	47	103	149	0.082	0.234
uS8	RPS22	0.020	0.003	0.070	0.017	0.215	0.034	0.336	0.082	872	149	1021	0.031	0.232
eS1	RPS1	0.013	0.002	0.036	0.008	0.137	0.016	0.174	0.040	3742	610	4352	0.015	0.142

Supplementary table 14: Structural superposition of r-proteins from *E.coli* and *S. cerevisiae*. Superposition of translating ribosomes from *E.coli* (PDB 5AFI) and *S. cerevisiae* (PDB 6NTU). Each individual r-protein from *S. cerevisiae* was investigated in regards of r-proteins from *E.coli* binding either at the same position or in close proximity with few aminoacids of spatial overlap. Eukaryote specific r-proteins that overlap with bacteria specific r-proteins are labelled in viollet.

		E. coli				E. coli	
r-protein	Yeast nom.	same position	close proximity	r-protein	Yeast nom.	same position	close proximity
uL1	RPL1	L1	-	eL6	RPL6	-	L21
uL16	RPL10	L16	-	uL30	RPL7	L30	-
uL5	RPL11	L5	-	eL8	RPL8	L9	-
uL11	RPL12	L11	-	uL6	RPL9	L6	-
eL13	RPL13	-	L15				
eL14	RPL14	-	L6, L13, L20	uS2	RPS0	S2	-
eL15	RPL15	L28	L9	eS1	RPS1	S6, S18	S11
uL13	RPL16	L13	-	eS10	RPS10	-	S3, S14
uL22	RPL17	L22	-	uS17	RPS11	S17	-
eL18	RPL18	-	L15, L4	eS12	RPS12	-	-
eL19	RPL19	-	-	uS15	RPS13	S15	-
uL2	RPL2	L2	-	uS11	RPS14	S11	-
eL20	RPL20	-	L25, L13	uS19	RPS15	S19	-
eL21	RPL21	L27, L25	-	uS9	RPS16	S9	-
eL22	RPL22	-	-	eS17	RPS17	-	S2
uL14	RPL23	L14	-	uS13	RPS18	S13	-
eL24	RPL24	L19	-	eS19	RPS19	-	S9, S13
uL23	RPL25	L23	-	uS5	RPS2	S5	-
uL24	RPL26	L24	-	uS10	RPS20	S10	-
eL27	RPL27	-	-	eS21	RPS21	-	S2, S8, S5
uL15	RPL28	L15	-	uS8	RPS22	S8	-
uL3	RPL3	L3	-	uS12	RPS23	S12	-
eL30	RPL30	-	L2	eS24	RPS24	-	S4, S16, S20
eL31	RPL31	L17, L32	-	eS25	RPS25	-	S7, S13
eL32	RPL32	-	L21	eS26	RPS26	S21	-
eL33	RPL33	L20, L13	L21	eS27	RPS27	-	S15, S8
eL34	RPL34	-	-	eS28	RPS28	-	S7
uL29	RPL35	L29	-	uS14	RPS29	S14	-
eL36	RPL36	-	L9, L15	uS3	RPS3	S3	-
eL37	RPL37	-	-	eS30	RPS30	-	S4, S12
eL38	RPL38	-	-	eS31	RPS31	-	-
eL39	RPL39	-	-	eS4	RPS4	S16	-
uL4	RPL4	L4	-	uS7	RPS5	S7	-
eL40	RPL40	-	-	eS6	RPS6	-	S20
eL42	RPL42	L33	-	eS7	RPS7	-	S8
eL43	RPL43	-	-	eS8	RPS8	S20	-
uL18	RPL5	L18	-	uS4	RPS9	S4	-

Supplementary Figures

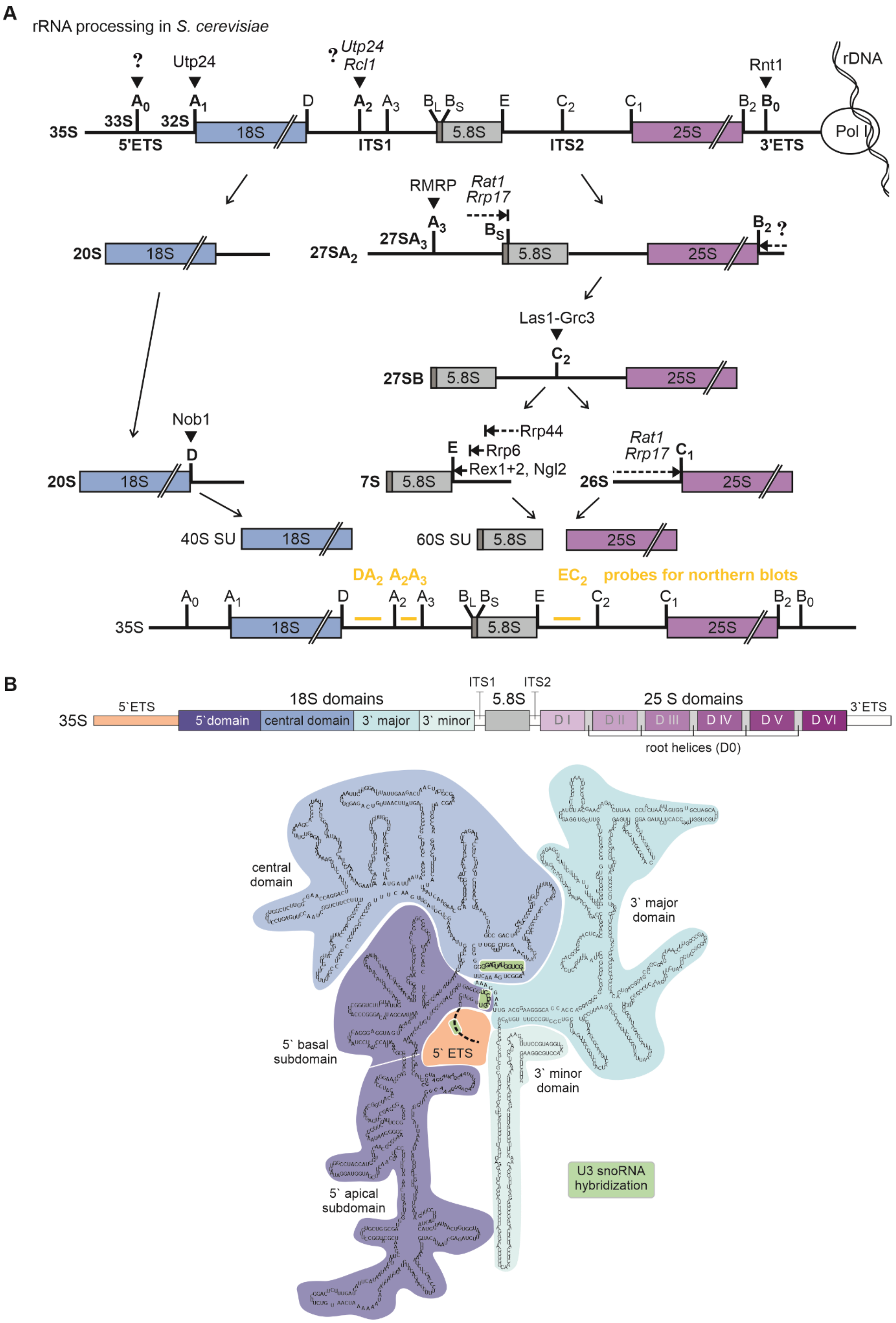

Supplementary Figure S1 (previous page): **A) Scheme of pre-rRNA processing in *S. cerevisiae.*** Exo- (--->) and endo- (▼) nucleolytic processing events during maturation of the 35S pre-rRNA. In the initial transcript, mature rRNAs (blue for the small, grey and purple for the large subunit rRNAs) are flanked by 5` and 3` external transcribed spacers (ETS) and separated by internal transcribed spacers 1 and 2 (ITS). Involved enzymes and resulting pre-rRNAs are indicated; questions marks and italic letters indicate uncertainties about the enzymes involved in processing the respective processing site. The last line shows the binding sites of the probes on the 35S pre-rRNA used for detection of different pre-rRNA species. **B) Domains of the 18S and 25S rRNA.** In the 35S pre-rRNA scheme the domains of the 18S rRNA are shown in blue colors. The domains of the 18S rRNA secondary structure are color coded accordingly.

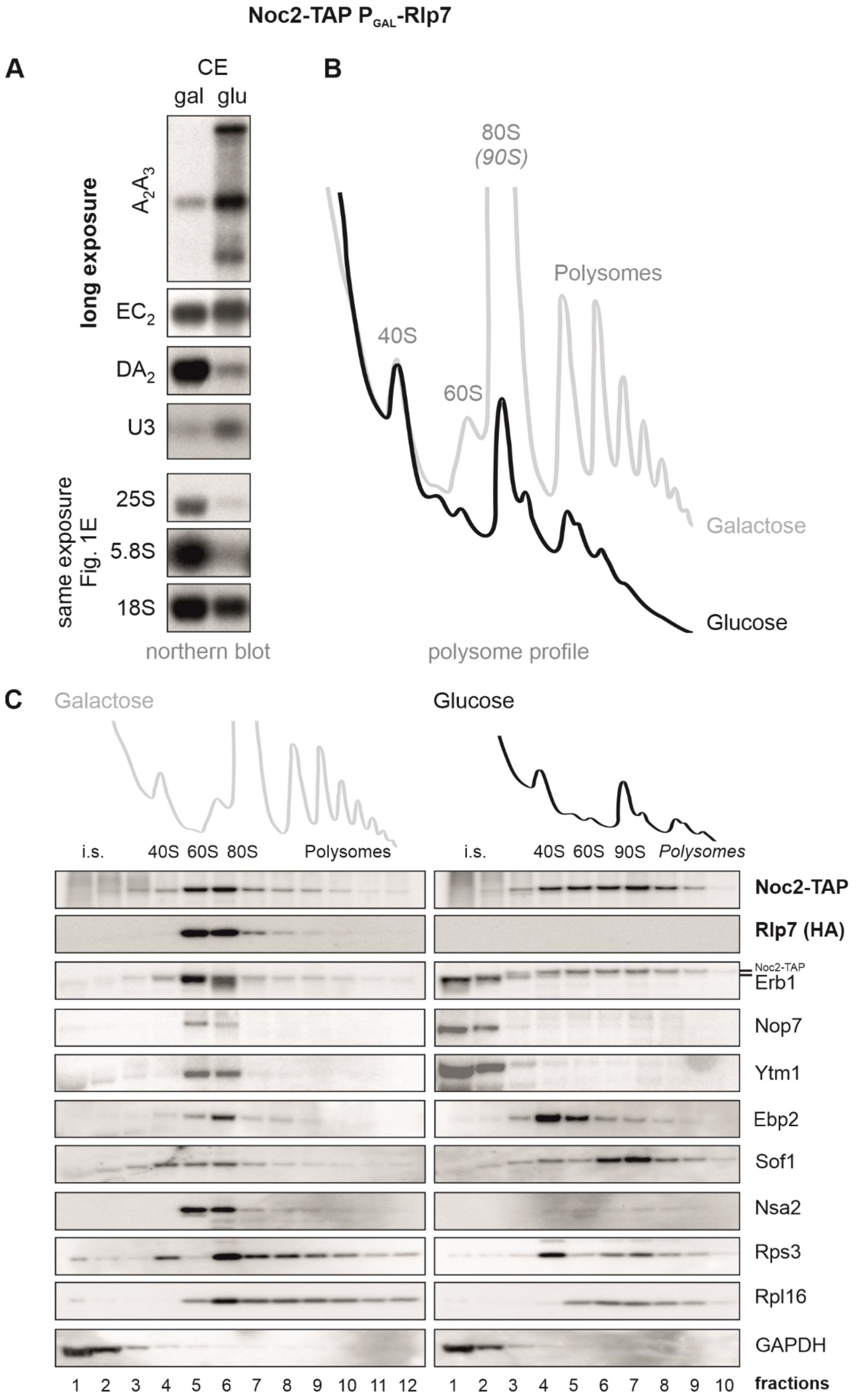

Supplementary Figure S2: **Depletion of Rlp7 rebounds to earlier steps in ribosome formation and leads to accumulation of a low molecular weight Nop7/Erb1/Ytm1 complex. A)** Northern blot. The strain expressing *RLP7* under the control of the *GAL1* promotor

was grown in galactose (gal; permissive condition) or glucose (glu; restrictive conditions) containing complete medium and pre-ribosomal particles were purified with Noc2-TAP and analyzed for pre-rRNA content in crude extracts and TEV eluate by northern blot. The exposures were adjusted to monitor changes of the RNA content in the crude extract (CE). Compare Figure 1E for exposures adjusted for eluates. **B)** Polysome profile analysis by sucrose gradient centrifugation. Comparison of the polysome profiles from Noc2-TAP *GAL1-RLP7* grown under permissive (grey line) and restrictive (black line) conditions. The strains were analyzed after 16 hours of depletion of Rlp7 and profiles were recorded by measuring the A260 over the pumped off volume. **C)** Sedimentation behavior of assembly factors. Fractions (1ml) from the sucrose gradient centrifugation were collected, proteins precipitated by TCA and analyzed by western blotting.

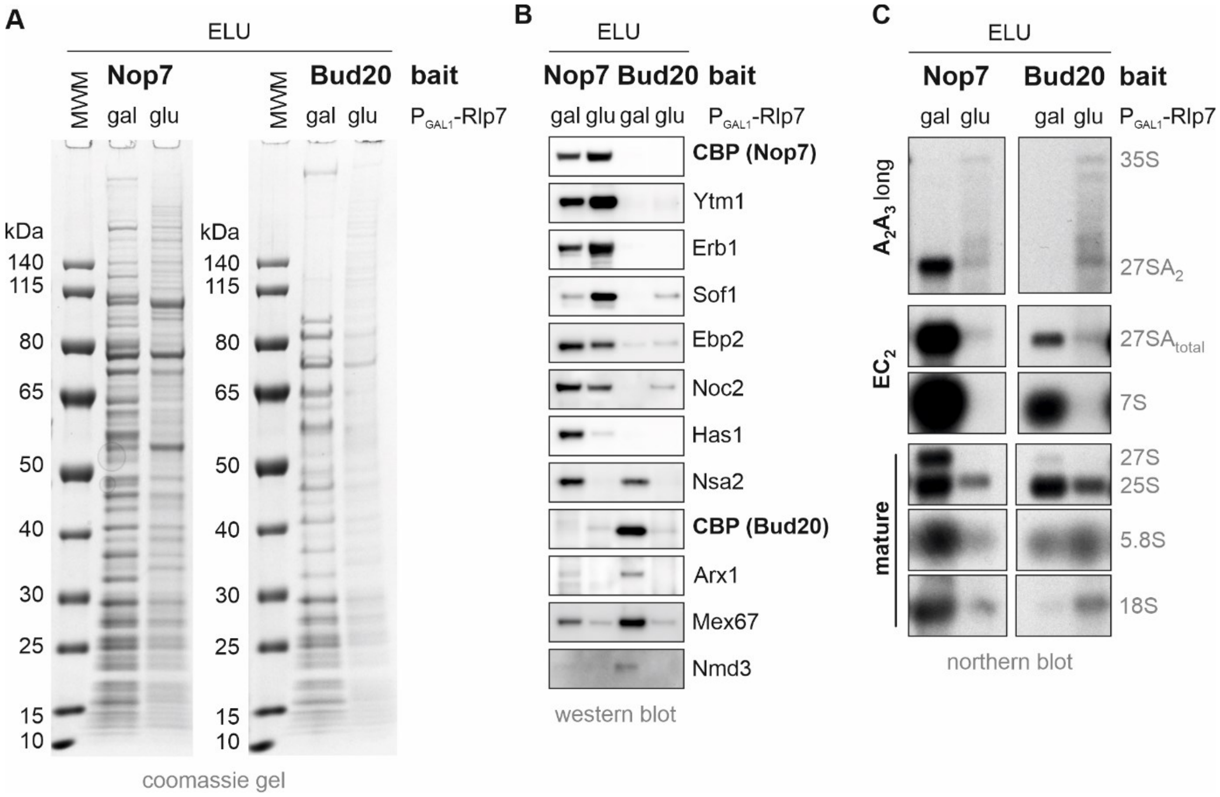

Supplementary Figure S3: **Effects of depletion of Rlp7 on downstream pre-ribosomal particles.** The strain expressing *RLP7* under the control of the *GAL1* promotor was grown in galactose (gal; permissive condition) or glucose (glu; restrictive conditions) containing complete medium and pre-ribosomal particles were purified with Nop7-TAP or Bud20-TAP as bait proteins. **A)** The isolated pre-ribosomes were analyzed by SDS-PAGE. **B)** The pre-ribosomes were analyzed by western blotting using antibodies directed to assembly and export factors. **C)** Northern blot showing RNAs co-purifying with Nop7-TAP and Bud20-TAP under permissive or restrictive conditions. The probes used are indicated on the left and the identified pre-rRNA species are indicated on the right. While under permissive conditions Nop7-TAP purifies a broad range of pre-60S ribosomal particles, it purifies a low molecular weight complex mainly comprised from Erb1, Nop7 and Ytm1 and lacking pre-rRNA under restrictive conditions (compare Supplementary Fig. S2C for the sedimentation behavior). In contrast, Bud20-TAP co-purifies late nuclear pre-60S particles under permissive conditions but might become degraded under restrictive conditions.

A non-depleted condition

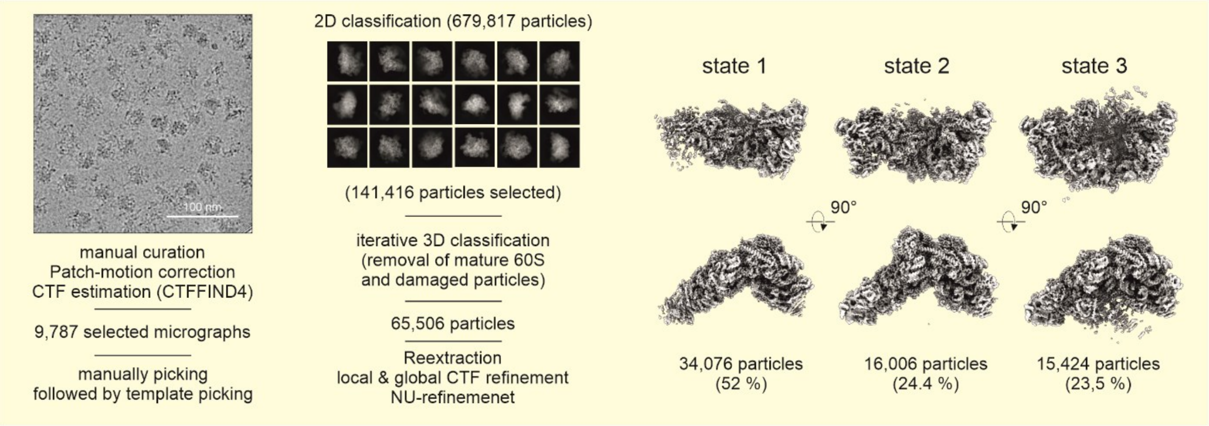

B Rlp7 depleted condition

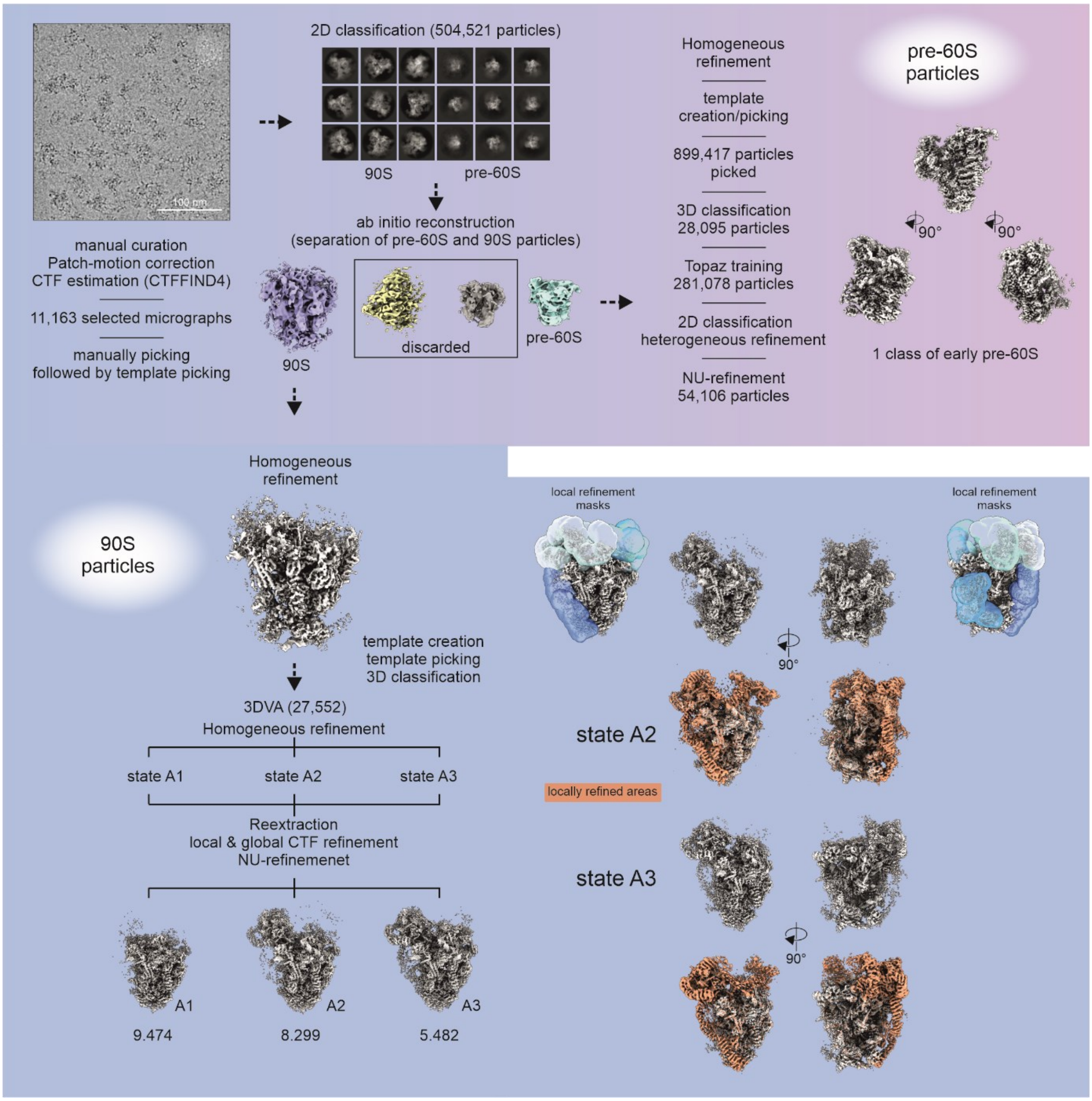

Supplementary Figure S4: **cryoEM processing scheme of Noc2-TAP particles. A)** Processing scheme for the pre-ribosomal particles isolated from the P_{GAL1}-*Rlp7* Noc2-TAP strain grown

under permissive conditions (galactose containing complete medium). **B)** Processing scheme for the pre-ribosomal particles isolated from the P_{GAL1}-*Rlp7* Noc2-TAP strain grown under restrictive conditions (glucose containing complete medium).

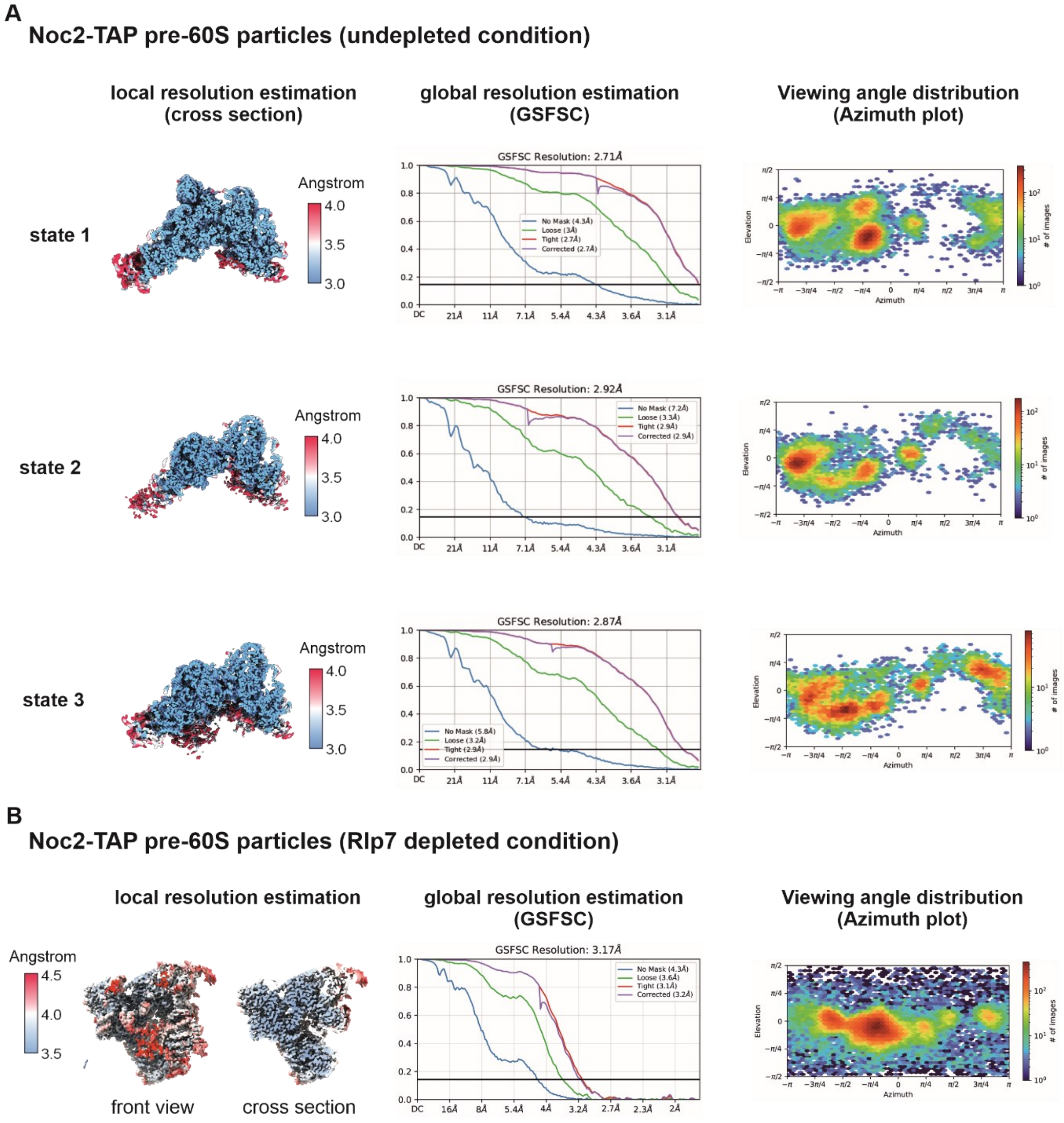

Supplementary Figure S5: **cryoEM reconstruction from pre-60S particles isolated from the PGAL1-RLP7 Noc2-TAP strain grown under permissive or restrictive conditions.** The maps were colored by their local resolution estimation. Global resolution estimation depicted by curves and viewing angle distribution as Azimuth plots are also shown. **A)** Pre-ribosomal particles purified via Noc2-TAP from the strain grown under permissive conditions showing sample states 1 to 3. The earliest state still contains the Nsa1 module (composed of Rpf1, Rrp1, Nop16 and Nsa1) and closely resembles state C of Kater et al. (Kater et al. 2017). The second state is almost identical, but lacks the Nsa1 module as the only difference. The third pre-60S structure closely represents state E of Kater et al. (Kater et al. 2017). **B)** Pre-ribosomal particles purified via Noc2-TAP from the strain grown under restrictive, Rlp7 depleted conditions

(growth in glucose containing medium). Only the pre-60S particle fraction is shown, for the 90S pre-ribosomal particle population see Supplementary Fig. S6.

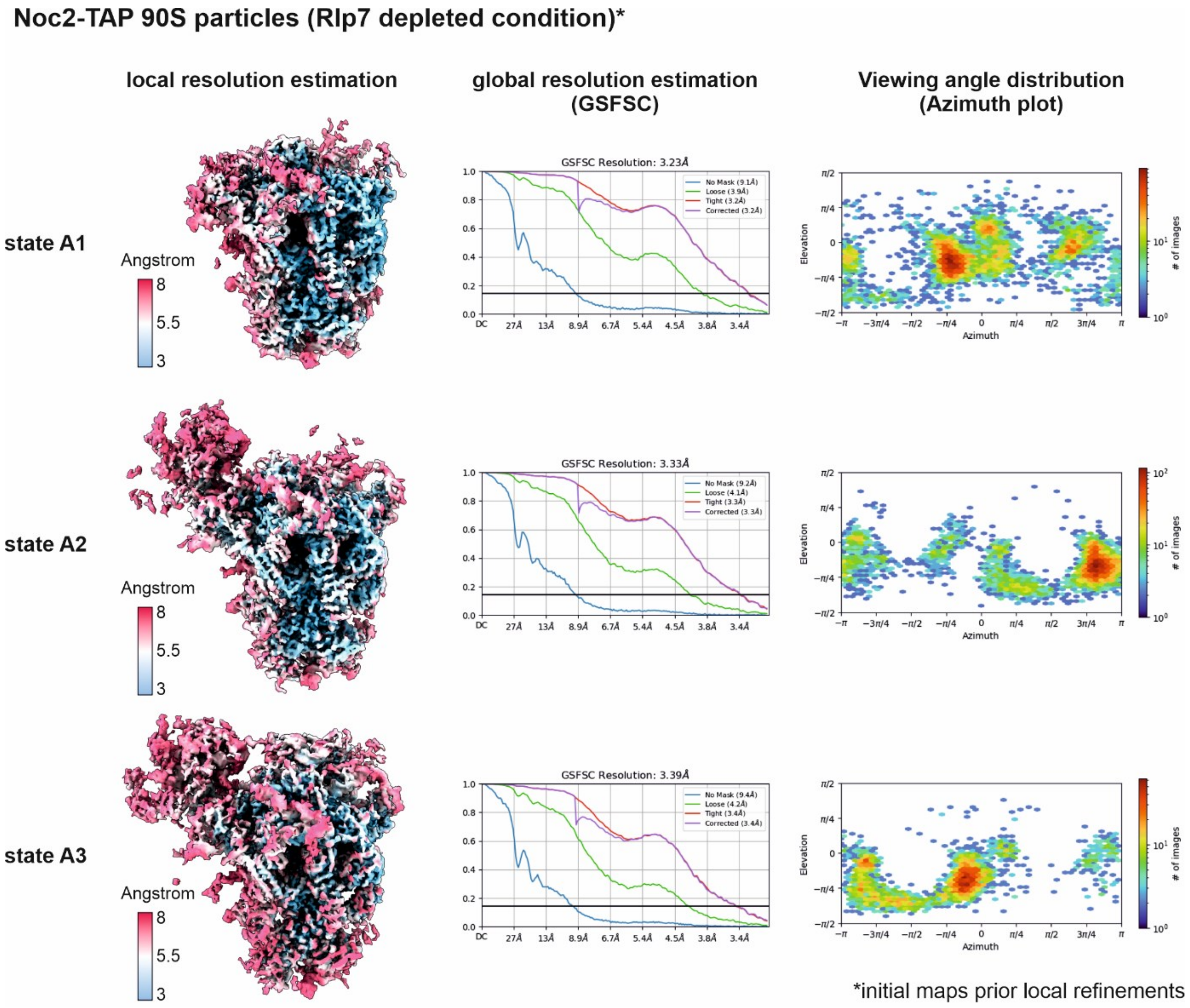

Supplementary Figure S6: **cryoEM reconstruction of three states from 90S particles isolated from the P_{GAL1}-RLP7 Noc2-TAP strain grown under restrictive conditions.** The maps were colored by their local resolution estimation. Global resolution estimation depicted by curves and viewing angle distribution as Azimuth plots are also shown.

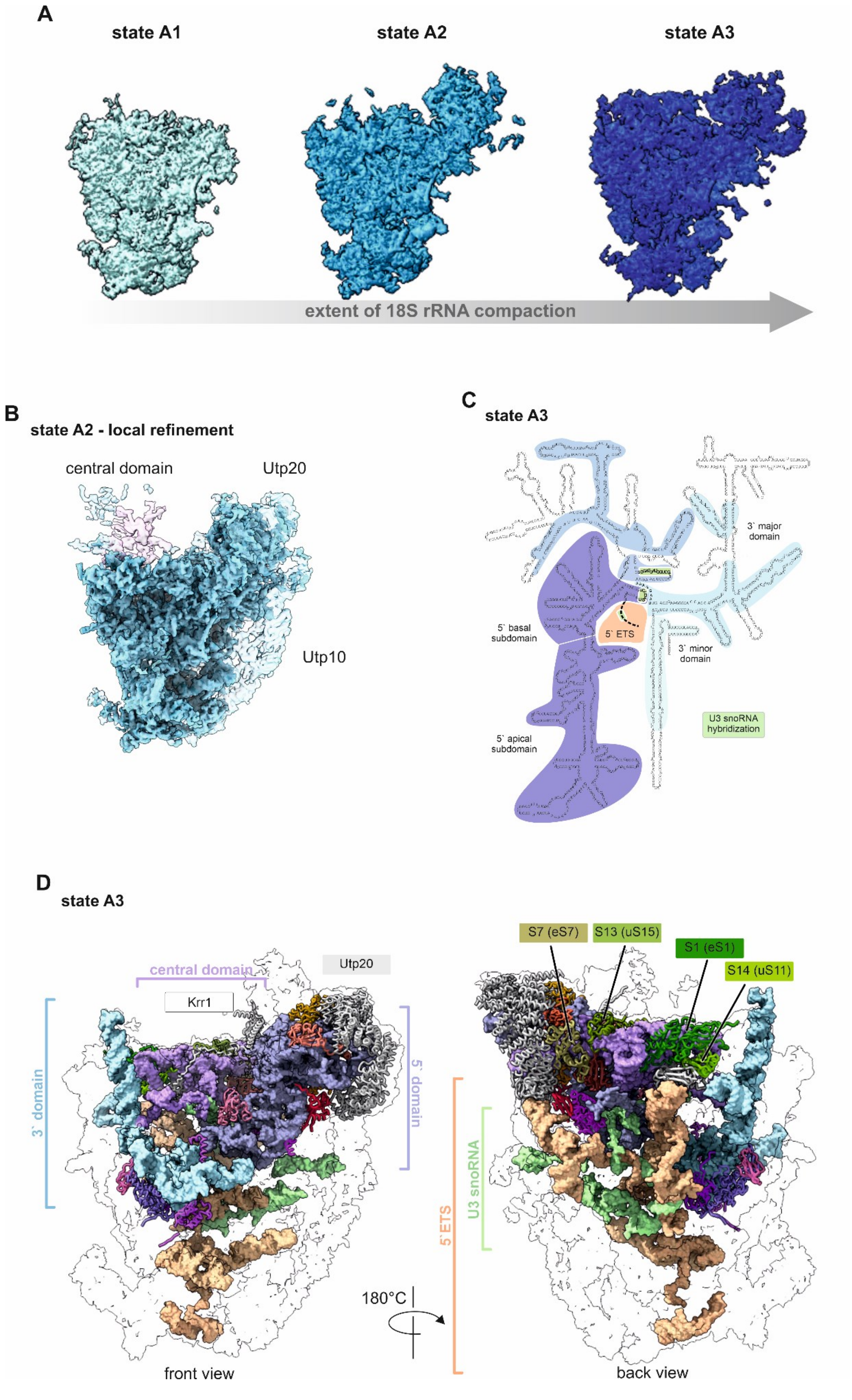
A
state A1
state A2
state A3
extent of 18S rRNA compaction
B
state A2 - local refinement
central domain
Utp20
Utp10
C
state A3
3` major domain
5` basal subdomain
5` ETS
3` minor domain
U3 snoRNA hybridization
5` apical subdomain
D
state A3
central domain
Utp20
Krr1
3` domain
5` domain
S7 (eS7)
S13 (uS15)
S1 (eS1)
S14 (uS11)
U3 snoRNA
5`ETS
180°C
front view
back view

Supplementary Figure S7 (previous page): **The observed state A3 closely represents the state B observed by the Ye lab** (Du et al. 2020) lacking Utp22, Rrp5, Rrp7, Enp1 and Rps27. **A)** Sequential order of the observed states of 90S particles. **B)** Local refinement of state A2 shows that the central domain of the 18S rRNA is in an immature, outwarded position. **C)** Colored regions within the three 18S rRNA domains indicate that they are structurally compacted in state A3. **D)** Front and back view on the state A3 90S particle. In addition to state A2 (Figure 3) the central domain is compacted and incorporated into the 90S particle together with the four ribosomal proteins labeled in green colors. R-proteins that are already incorporated at earlier timepoints are colored the same as in figure 2.

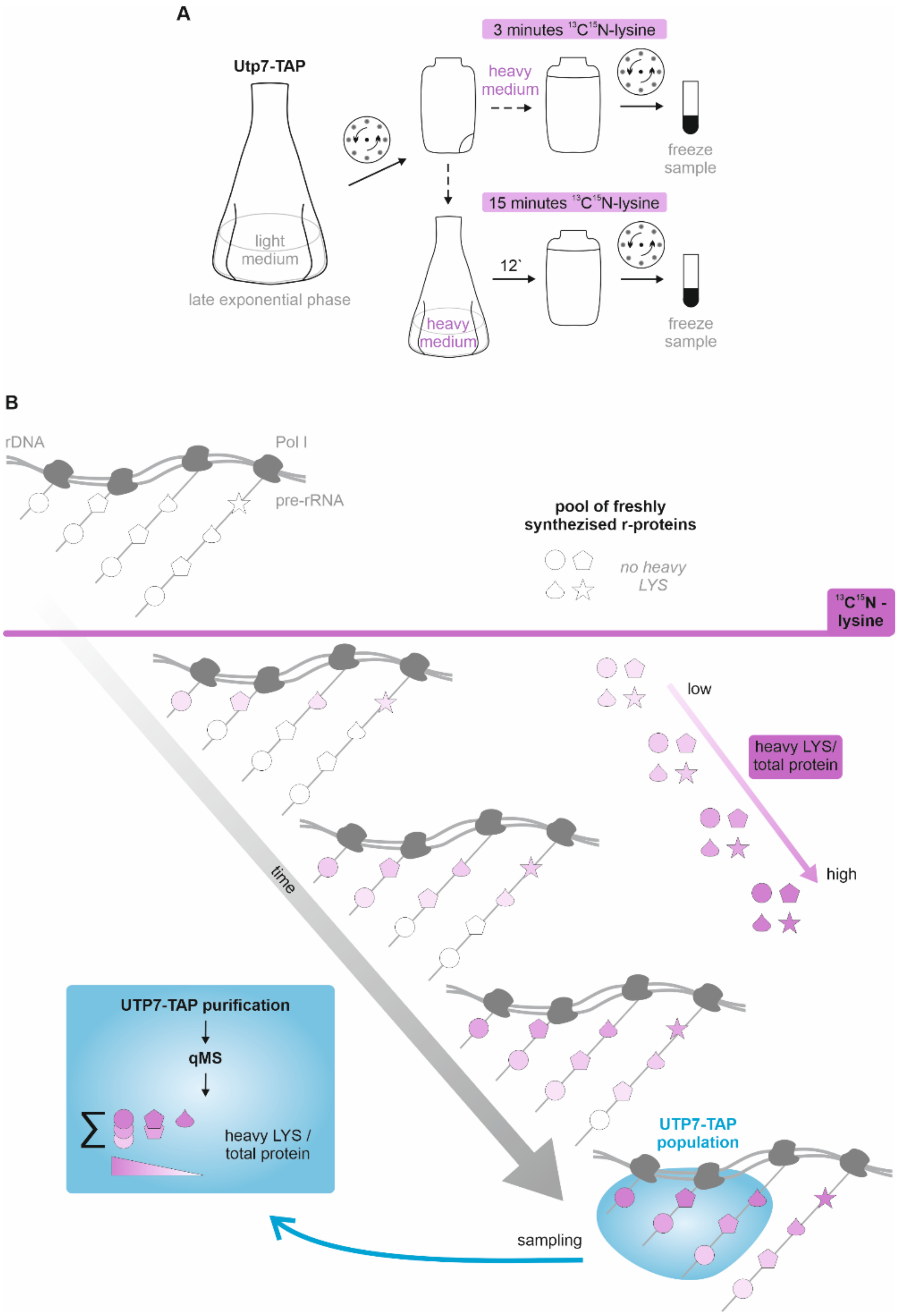

Supplementary Figure S8: **Scheme of stable isotope labeling by amino acids in cell culture (SILAC). A)** The Utp7-TAP strain was grown to late exponential phase in SD media containing

unlabeled amino acids (light medium). After centrifugation (on room temperature) cells were either resuspended in $^{13}C^{15}N$-lysine containing SD medium (heavy media) and subsequently centrifuged for the 3 minutes sample. In a second approach, cells were transferred to a flask with $^{13}C^{15}N$-lysine containing SD medium, incubated for 12 minutes followed by centrifugation by 3 minutes (15 minutes sample). **B)** Upon incubation in heavy medium ribosomal proteins will be labeled with $^{13}C^{15}N$-lysine, with early incorporated ribosomal proteins showing higher labels in TAP purifications of very early pre-ribosomal particles than later incorporated proteins. This can be measured by qMS comparing the labeled peptide intensities to the total peptide intensities.

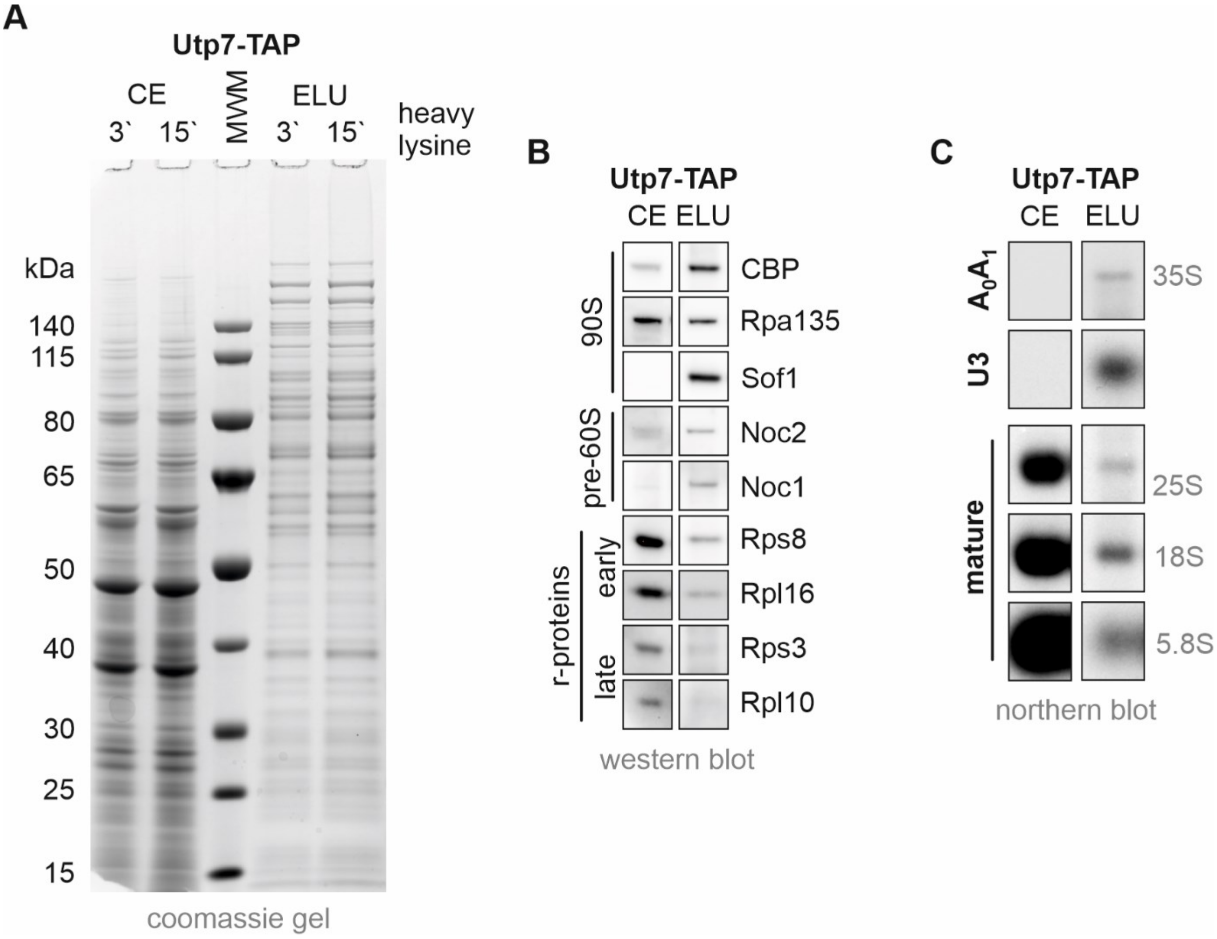

Supplementary Figure S9: **Purification of Utp7-TAP for SILAC measurement. A)** Coomassie stained gel of the SILAC samples labeled with heavy lysine ($^{13}C^{15}N$-lysine) for either three or fifteen minutes (3` / 15`) and purified via Utp7-TAP. Crude extract as well as eluate samples are shown. **B & C)** Unlabeled control (regular minimal media) purification of Utp7-TAP with western blot **(B)** and northern blot **(C)** analysis of the crude extract and the eluate to verify that early pre-ribosomal particles are isolated.

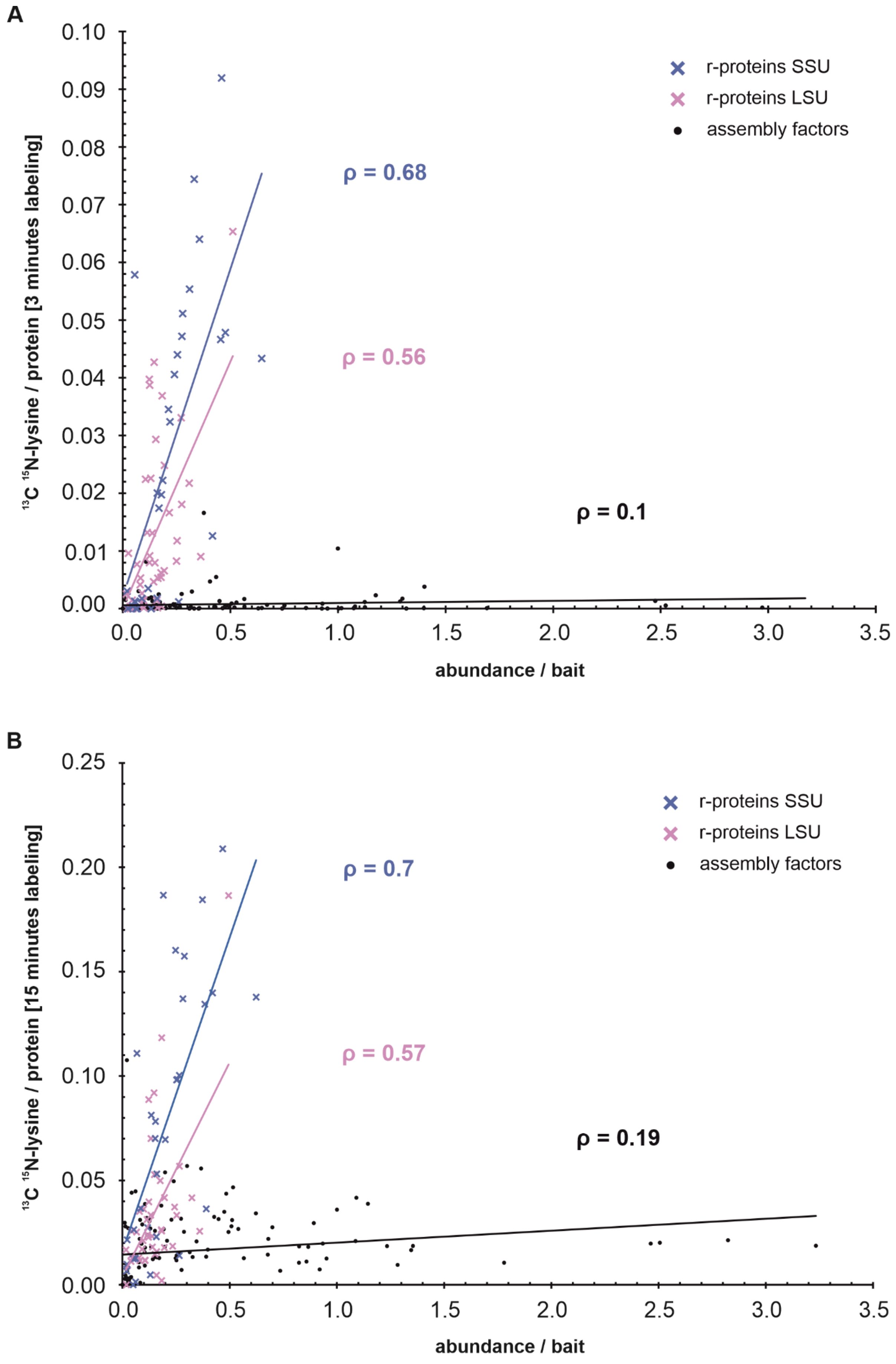

A
0.10
0.09
0.08
0.07
0.06
0.05
0.04
0.03
0.02
0.01
0.00
0.0
0.5
1.0
1.5
2.0
2.5
3.0
3.5
^{13}C ^{15}N-lysine / protein [3 minutes labeling]
abundance / bait
r-proteins SSU
r-proteins LSU
assembly factors
ρ = 0.68
ρ = 0.56
ρ = 0.1
B
0.25
0.20
0.15
0.10
0.05
0.00
0.0
0.5
1.0
1.5
2.0
2.5
3.0
3.5
^{13}C ^{15}N-lysine / protein [15 minutes labeling]
abundance / bait
r-proteins SSU
r-proteins LSU
assembly factors
ρ = 0.7
ρ = 0.57
ρ = 0.19

Supplementary Figure S10 (previous page): **Correlation between abundance to the bait and the relative label with heavy lysine.** For the 3 minutes **(A)** as well as the 15 minutes **(B)** qMS sample sets the abundance to the bait (Utp7) was blotted against the relative amount of heavy labeled lysine, for the individual proteins. Ribosomal proteins of the small subunit (SSU) are colored blue, ribosomal proteins of the large subunit (LSU) are labeled in lavender, while assembly factors are colored black. For each dataset the Pearson correlation between the abundance to the bait and the relative label was calculated and is given as ρ (-1 negative linear correlation, 0 = no correlation, +1 = positive linear correlation). n =3 (mean values are shown).

References

Ban, Nenad; Beckmann, Roland; Cate, Jamie H. D.; Dinman, Jonathan D.; Dragon, François; Ellis, Steven R. et al. (2014): A new system for naming ribosomal proteins. In: *Current opinion in structural biology* 24, S. 165–169. DOI: 10.1016/j.sbi.2014.01.002.

Du, Yifei; An, Weidong; Zhu, Xing; Sun, Qi; Qi, Jia; Ye, Keqiong (2020): Cryo-EM structure of 90S small ribosomal subunit precursors in transition states. In: *Science (New York, N.Y.)* 369 (6510), S. 1477–1481. DOI: 10.1126/science.aba9690.

Gerhalter, Magdalena; Kofler, Lisa; Zisser, Gertrude; Merl-Pham, Juliane; Hauck, Stefanie M.; Bergler, Helmut (2024): The novel pre-rRNA detection workflow "Riboprobing" allows simple identification of undescribed RNA species. In: *RNA (New York, N.Y.)*. DOI: 10.1261/rna.079912.123.

Janke, Carsten; Magiera, Maria M.; Rathfelder, Nicole; Taxis, Christof; Reber, Simone; Maekawa, Hiromi et al. (2004): A versatile toolbox for PCR-based tagging of yeast genes: new fluorescent proteins, more markers and promoter substitution cassettes. In: *Yeast (Chichester, England)* 21 (11), S. 947–962. DOI: 10.1002/yea.1142.

Kater, Lukas; Thoms, Matthias; Barrio-Garcia, Clara; Cheng, Jingdong; Ismail, Sherif; Ahmed, Yasar Luqman et al. (2017): Visualizing the Assembly Pathway of Nucleolar Pre-60S Ribosomes. In: *Cell* 171 (7), 1599-1610.e14. DOI: 10.1016/j.cell.2017.11.039.
